# Applied causal inference methods for sequential mediators

**DOI:** 10.1186/s12874-022-01764-w

**Published:** 2022-11-24

**Authors:** D Zugna, M Popovic, F Fasanelli, B Heude, G Scelo, L Richiardi

**Affiliations:** 1 Cancer Epidemiology Unit, Department of Medical Sciences, University of Turin, Via Santena 7, 10126 Turin, Italy; 2 Université de Paris Cité, Inserm, INRAE, Centre of Research in Epidemiology and StatisticS (CRESS), F-75004 Paris, France

**Keywords:** Causal inference, Mediation analysis, Sequential mediators, Direct and indirect effects, Weighting, Imputation

## Abstract

**Background:**

Mediation analysis aims at estimating to what extent the effect of an exposure on an outcome is explained by a set of mediators on the causal pathway between the exposure and the outcome. The total effect of the exposure on the outcome can be decomposed into an indirect effect, i.e. the effect explained by the mediators jointly, and a direct effect, i.e. the effect unexplained by the mediators. However finer decompositions are possible in presence of independent or sequential mediators.

**Methods:**

We review four statistical methods to analyse multiple sequential mediators, the inverse odds ratio weighting approach, the inverse probability weighting approach, the imputation approach and the extended imputation approach. These approaches are compared and implemented using a case-study with the aim to investigate the mediating role of adverse reproductive outcomes and infant respiratory infections in the effect of maternal pregnancy mental health on infant wheezing in the Ninfea birth cohort.

**Results:**

Using the inverse odds ratio weighting approach, the direct effect of maternal depression or anxiety in pregnancy is equal to a 59% (95% CI: 27%,94%) increased prevalence of infant wheezing and the mediated effect through adverse reproductive outcomes is equal to a 3% (95% CI: -6%,12%) increased prevalence of infant wheezing. When including infant lower respiratory infections in the mediation pathway, the direct effect decreases to 57% (95% CI: 25%,92%) and the indirect effect increases to 5% (95% CI: -5%,15%). The estimates of the effects obtained using the weighting and the imputation approaches are similar. The extended imputation approach suggests that the small joint indirect effect through adverse reproductive outcomes and lower respiratory infections is due entirely to the contribution of infant lower respiratory infections, and not to an increased prevalence of adverse reproductive outcomes.

**Conclusions:**

The four methods revealed similar results of small mediating role of adverse reproductive outcomes and early respiratory tract infections in the effect of maternal pregnancy mental health on infant wheezing. The choice of the method depends on what is the effect of main interest, the type of the variables involved in the analysis (binary, categorical, count or continuous) and the confidence in specifying the models for the exposure, the mediators and the outcome.

**Supplementary Information:**

The online version contains supplementary material available at 10.1186/s12874-022-01764-w.

## Background

Mediation analysis aims at estimating to what extent the effect of an exposure on an outcome is explained by a given set of mediators on the causal pathway between the exposure and the outcome. This goal is achieved by decomposing the total effect of the exposure on the outcome into a natural indirect effect, i.e. the effect explained through the given mediators, and a natural direct effect, i.e. the effect unexplained by the mediators [1]. Researchers often deal with research questions that involve more than one mediator at a time. In life course epidemiology it is often important to elucidate the processes that link early life factors to later health. In most cases these involve multiple mechanisms which identification in many instances implies possible interventions with a consequent impact on public health. For example, one could be interested in understanding the mediating role of breastfeeding in the first year of life and diet after the first year of life in the effect of socio-economic status on obesity in infancy, or the mediating role of lower respiratory tract infections and infant wheezing in the effect of day care attendance on asthma at school age.

In the analysis of multiple mediators, the total effect of the exposure on the outcome can be decomposed into the natural direct and indirect effects considering the mediators jointly. As the number of mediators increases, so does the difficulty to estimate these effects. The estimating model assumptions vary according to the approach used to estimate these effects, in particular their complexity increases when the method requires the specification of the joint density for the mediators. Such complexity further increases when finer decompositions of the total effect are of interest; in a setting with two mediators, for example, there are four possible pathways from the exposure to the outcome: through the first mediator alone, through the second mediator alone, through both mediators, and through neither of them. When the aim is to decompose the total effect of the exposure on the outcome as the sum of separate effects along each of the possible pathways, the number of assumptions and models that should be specified to identify and estimate each of the effects increases with increasing number of mediators [2].

The counterfactual approach provides a set of tools to identify and estimate direct and indirect effects using both linear and nonlinear models, with both discrete and continuous variables, and allowing interactions between the exposure and the mediators [1, 3–6]. Furthermore it clearly specifies the assumptions needed to identify the direct and indirect effects and to allow their causal interpretation [1, 7–10].

A number of methods, which derive from different characterisation of the non-parametric mediation formula [3], based on the counterfactual framework, have been developed to carry out mediation analysis involving multiple mediators. According to the research question, there are i) methods that address the problem related to exposure-induced mediator-outcome confounders in estimating the indirect effect through a mediator of primary interest, ii) methods that aim at estimating the indirect effect through multiple mediators jointly and iii) methods that aim at estimating the separate effects along each of the possible mediation pathways. Imai et al (2010) [11] proposed a quasi-Bayesian Monte Carlo method, or alternatively, a nonparametric bootstrap procedure, to draw counterfactuals from the outcome and mediators models and hence calculate the indirect effects through the mediator of primary interest irrespective of whether they also come through the alternative mediators. Vanderweele and Vansteenlandt [12] proposed a regression-based approach to estimate the indirect effect through mediators jointly using a combination of regression parameters obtained from models for the mediators and the outcome. This approach can be used only in the context of continuous outcomes and continuous or binary mediators, or rare and binary outcomes with continuous mediators. To overcome these limits, Vanderweele and Vansteenlandt [12] presented an alternative approach based on inverse probability weighting (IPW) that can be used for any type of outcome, including non-rare binary outcomes, and does not require to specify any model for the mediators. Similarly, the inverse odds ratio weighting approach (IOR) proposed by Tchetgen Tchetgen [13, 14] and the imputation approach developed by Vansteelandt et al [15] can be fitted to multiple scenarios, with different types of exposure, mediators and outcome without the necessity to model the distribution of the mediators. Steen et al [16] proposed an extension of the imputation approach to estimate not only the indirect effect through mediators jointly but also the separate effects along some specific mediation pathways. Daniel at al (2015) [2] extended the parametric G-computation [17, 18] to the context of multiple mediators by Monte Carlo simulation allowing the decomposition into multiple path-specific effects through many mediators. Albert et al (2019) [19] showed a further development of the parametric mediation formula approach to accomodate repeatedly measured mediators and multiple mediators at each stage and allow for multiple types of outcomes following generalized linear models. Being based on parametric models, the approaches described above provide valid estimates when all models are correctly specified.

In this paper, we provide a detailed overview and step-by-step implementation with the statistical software R [20] of four methods to analyse sequential mediators: the inverse odds ratio weighting approach (IOR) [13, 14], the inverse probability weighting approach (IPW) [12], the imputation approach [15] and the extended imputation approach [16]. Even if these methods differ for what regards the estimation procedure, they share several similarities including the possibility to consider binary, categorical or continuous exposures, the possibility to model any type of outcome through generalised linear models and the non-necessity to specify a regression model for the distribution of the mediators. Although the parametric Monte Carlo approach proposed by Daniel et al (2015) [2] provides a finer decomposition of the total effect than the four selected methods, it also requires to model the joint distribution of the mediators. Hence the non-necessity to model the mediators jointly is a strength of the selected methods.

The paper is organised as follows: (i) we introduce the case-study of interest, (ii) we introduce the framework and notations, (iii) we describe the selected approaches to the analysis of multiple mediators, (iv) we apply the methods to the case-study and (v) we discuss the results.

## Case study

Infant wheezing is a frequent condition in the first two years of life with a prevalence of more than 30% in European countries [21]. As wheezing in early life is one of the strongest determinants of later childhood asthma, disentangling its aetiology and mechanisms is a priority in asthma research. There is growing evidence of a relationship between antenatal maternal psychological distress and development of child wheeze, but the mechanisms underlying this association are still unclear. Our aim is to investigate the mediating role of adverse reproductive outcomes and infant respiratory infections underlying the effect of maternal mental health during pregnancy on infant wheezing between 6 and 18 months. Hence, we focus on two potential sequential mechanisms, through the adverse reproductive outcomes and then infant respiratory infections.

**Fig. 1:**
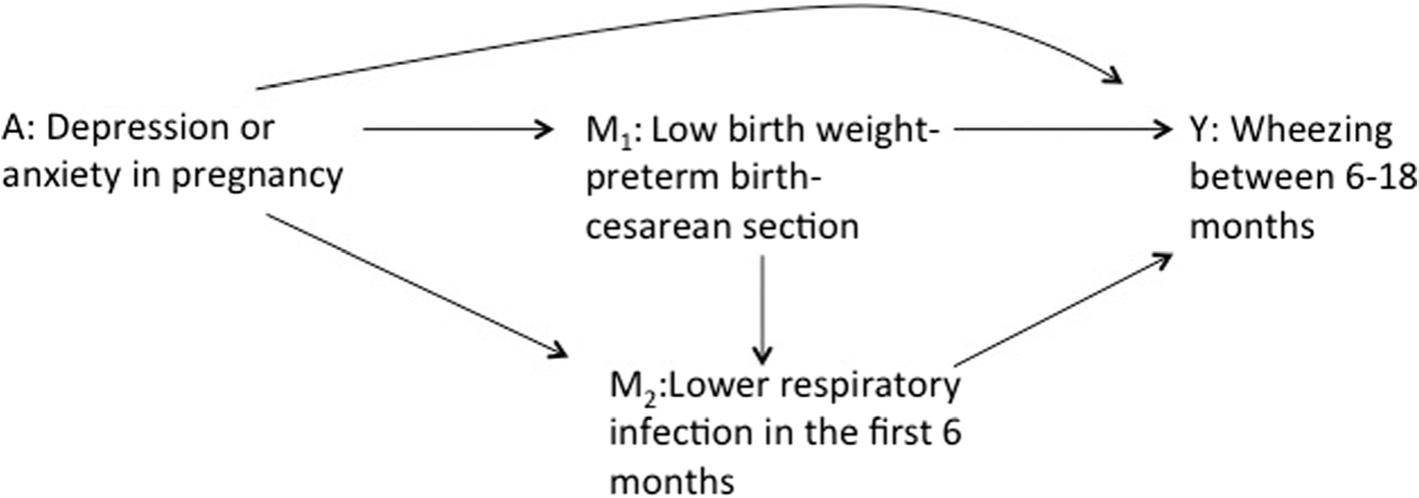
DAG representing the hypothesized causal structure of the case study. For the sake of simplicity the confounders C are not shown

We use data from 4797 infants of the Ninfea cohort [22]. Ninfea is a web-based birth cohort with the aim of investigating the effects of early-life exposures on the health of newborns, children, adolescents, and adults. Cohort members are children of mothers recruited between 2005 and 2016 in Italy who completed a first online questionnaire at any time during their pregnancy and are invited to complete six follow-up questionnaires when their child turn 6 months, 18 months, 4, 7, 10 and 13 years of age. The study was approved by the local Ethical Committee (project n. 45). Informed consent was obtained from all the participants. We consider a binary exposure *A* indicating whether or not the woman had depression or anxiety in pregnancy; a binary mediator $M_{1}$ that indicates the occurrence of at least one between low birth weight, preterm birth, or delivery with cesarean section (hereafter collectively referred as to “adverse reproductive outcomes”); a binary mediator $M_{2}$ for the occurrence of lower respiratory infections in the first 6 months of infant life, as reported at the 6-month follow-up questionnaire; and an outcome *Y* for the occurrence of wheezing between 6 and 18 months of infant life, as reported at the 18-month follow-up questionnaire. Maternal age, education, region of residence, and pre-pregnancy body mass index, parity and child’s sex are considered as baseline confounders *C*. Although the example is necessarily simplified, we assume that the selected set of confounders is sufficient to satisfy the assumptions which will be defined in the Assumptions Section. The underlying hypothesized causal structure is represented in Fig. 1, in which $M_{1}$ and $M_{2}$ are assumed to be sequential and the confounders C are not shown for the sake of simplicity. The variables involved in the analysis are described in the Supplemental Material.

## Methods

### Marginal and conditional effects

**Fig. 2:**
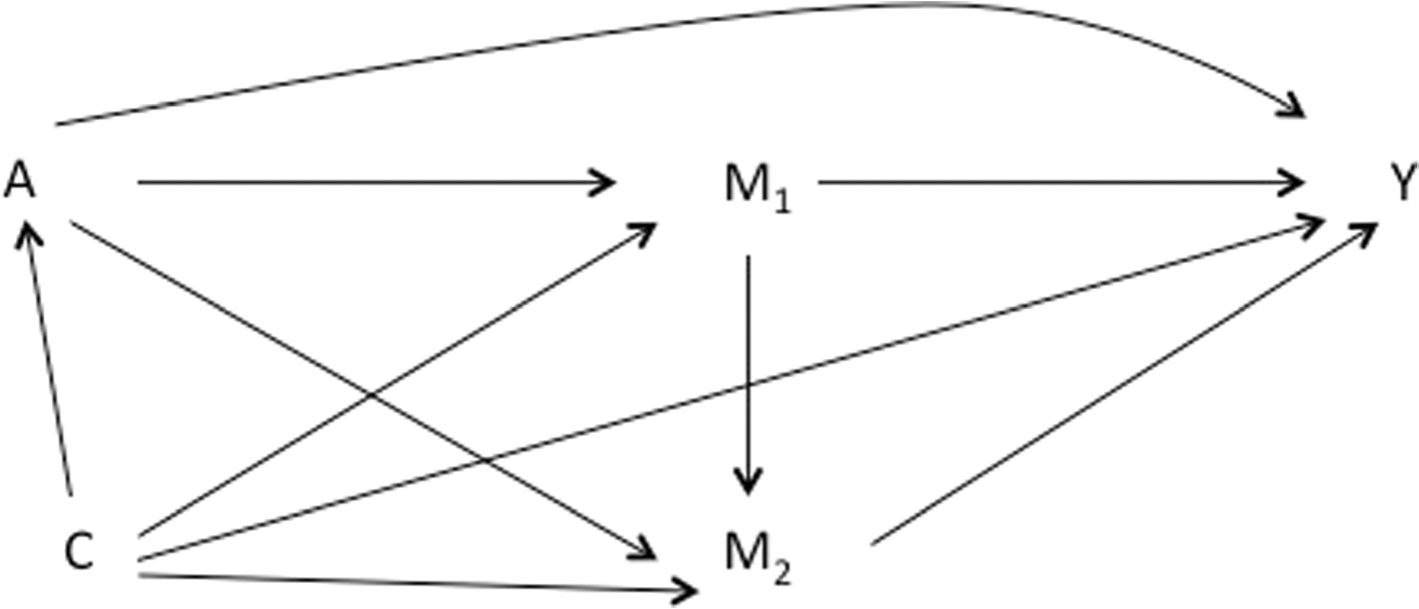
DAG representing the hypothesized causal structure. A: exposure, M_1_: first mediator, M_2_: second mediator, Y: outcome, C: confounders of A-Y, A-M_1_, A-M_2_, M_1_-Y, M_2_-Y, M_1_-M associations

We consider a setting with two sequential mediators. Let *A* denote the exposure, *Y* denote the outcome, and $M_{1}$ and $M_{2}$ denote two potential mediators on the pathway from the exposure to the outcome (with *A* affecting both $M_{1}$ and $M_{2}$, and $M_{1}$ affecting $M_{2}$). Let *C* denote the set of confounders that may affect the exposure, the mediators and/or the outcome. The relationships between *A*, $M_{1}$, $M_{2}$, *Y* and *C* are represented in the Directed Acyclic Graph (DAG) shown in Fig. 2. Let $Y(a, M_{1}(a^{*}),M_{2}(a^{*},M_{1}(a^{*})))$ be the individual counterfactual outcome that would have been observed had the exposure *A* been set to *a* and had $M_{1}$ and $M_{2}$ been set to the natural value they would have taken if *A* had been $a^{*}$, where *a* and $a^{*}$ denote two possible exposure levels (e.g. $a=1$ and $a^{*}=0$).

In the case-study, *a* and $a^{*}$ correspond to the levels of the variable depression or anxiety in pregnancy (presence *vs* absence); $M_{1}(a^{*})$ to the level of the adverse reproductive outcomes (presence *vs* absence) that would have been observed had the mother not suffered from depression or anxiety in pregnancy (if *A* were set to $a^{*}$); $M_{2}(a^{*},M_{1}(a^{*}))$ to the level of the occurrence of lower respiratory infections (presence *vs* absence) that would have been observed if the mother had not suffered from depression or anxiety in pregnancy (if *A* were set to $a^{*}$) and the adverse reproductive outcomes were set to the level that would have been observed if the mother had not suffered from depression or anxiety in pregnancy (*A* were set to $a^{*}$). $Y(a, M_{1}(a^{*}),M_{2}(a^{*},M_{1}(a^{*}))$ corresponds to the occurrence of wheezing between 6 and 18 months of infant life (presence *vs* absence) that would have been observed if i)the mother had suffered from depression or anxiety in pregnancy (*A* set to *a*); ii)the adverse reproductive outcomes were set to the level that would have been observed if the mother had not suffered from depression or anxiety in pregnancy (*A* set to $a^{*}$); and iii)the occurrence of lower respiratory infections were set to the level that would have been observed if the mother had not suffered from depression or anxiety in pregnancy (*A* set to $a^{*}$) and the adverse reproductive outcomes were set to the level that would have been observed if the mother had not suffered from depression or anxiety in pregnancy (*A* set to $a^{*}$). Finally $Y(a, M_{1}(a),M_{2}(a^{*},M_{1}(a))$ corresponds to the level of the occurrence of wheezing between 6 and 18 months of infant life that would have been observed if i)the mother had suffered from depression or anxiety in pregnancy (*A* set to *a*); ii)the adverse reproductive outcomes were set to the level that would have been observed if the mother had suffered from depression or anxiety in pregnancy (*A* set to *a*); and iii)the occurrence of lower respiratory infections were set to the level that would have been observed if the mother had not suffered from depression or anxiety in pregnancy but the adverse reproductive outcomes were set to the level that would have been observed if the mother had suffered from depression or anxiety in pregnancy.

At the population level the marginal total effect of *A* on *Y* can be decomposed with respect to the joint mediator $\{M_{1},M_{2}\}$ as follows:

1 $$\begin{aligned}&\overbrace{g\{E[Y(a, M_{1}(a),M_{2}(a,M_{1}(a)))]\}-g\{E[Y(a^{*}, M_{1}(a^{*}),M_{2}(a^{*},M_{1}(a^{*})))]\}}^\text {Marginal total effect}= \end{aligned}$$

2 $$\begin{aligned}&\overbrace{g\{E[Y(a, M_{1}(a),M_{2}(a,M_{1}(a)))]\}-g\{E[Y(a, M_{1}(a^{*}),M_{2}(a^{*},M_{1}(a^{*})))]\}}^\text {Marginal natural indirect effect}+ \end{aligned}$$

3 $$\begin{aligned}&\overbrace{g\{E[Y(a, M_{1}(a^{*}),M_{2}(a^{*},M_{1}(a^{*})))]\}-g\{E[Y(a^{*}, M_{1}(a^{*}),M_{2}(a^{*},M_{1}(a^{*})))]\}}^\text {Marginal natural direct effect} \end{aligned}$$

where *g* is a link function [1]. For example, if the scale is linear the link function is the identity function, if the scale is odds ratio the link function is the logit function. Note that if the scale is additive (for example odds ratio on logarithmic scale) the total effect equals the sum of the natural direct and natural indirect effects, while, if the scale is multiplicative (for example odds ratio), the total effect equals the product of those two effects. The formula above states that the marginal total effect (1) can be decomposed into two components, the marginal natural indirect effect that acts through at least one of the mediators (2) and the marginal natural direct effect that does not involve any of the mediators (3). Finest decompositions of the total effect into a direct effect of the exposure on the outcome and an indirect effect operating through a number of possible pathways have been proposed [2] including also the decomposition of the total effect into the effects that are due to mediation only, interaction only, both mediation and interaction, neither mediation nor interaction (two-way and three-way interactions) [23, 24].

The marginal total effect expresses how much the outcome would change (on the scale defined by *g*) if the exposure were set from level $a^{*}$ to level *a* uniformly in the population. The marginal natural direct effect expresses how much the outcome would change if the exposure were set at $A=a$ versus $A=a^{*}$ but both mediators were kept at the level they would have naturally taken had the exposure been set at $A=a^{*}$. Thus this effect captures the remaining effect of the exposure on the outcome if we were able to disable the pathways from the exposure to the mediators. The marginal natural indirect effect expresses how much the outcome would change if the exposure were fixed at the level $A=a$ but both mediators were changed from the level they would have taken if $A=a^{*}$ to the level they would have taken if $A=a$. Thus this estimand captures the effect of the exposure on the outcome that operates through the mediators jointly.

In the case-study, the marginal total effect expresses how much the occurrence of wheezing between 6 and 18 months of infant life would differ when comparing two hypothetical scenarios in which all women suffered from depression or anxiety in pregnancy versus all women did not suffer from depression or anxiety in pregnancy. The marginal natural direct effect expresses how much the occurrence of wheezing between 6 and 18 months of infant life would differ comparing two hypothetical scenarios in which all women suffered from depression or anxiety in pregnancy versus all women did not suffer from depression or anxiety in pregnancy but both adverse reproductive outcomes and occurrence of lower respiratory infections were kept at the level they would have naturally taken in absence of depression or anxiety in pregnancy. The marginal natural indirect effect expresses how much the occurrence of wheezing between 6 and 18 months of infant life would differ if women suffered from depression or anxiety in pregnancy but both adverse reproductive outcomes and occurrence of lower respiratory infections were shifted from the level they would have taken if women did not suffer from depression or anxiety in pregnancy to the level they would have taken if women suffered from depression or anxiety in pregnancy.

Alternatively the total, natural direct, and natural indirect effects can be defined conditionally on a set of baseline confounders *C* as follows:

4 $$\begin{aligned}&\overbrace{g\{E[Y(a, M_{1}(a),M_{2}(a,M_{1}(a)))\vert C=c]\}-g\{E[Y(a^{*}, M_{1}(a^{*}),M_{2}(a^{*},M_{1}(a^{*})))\vert C=c]\}}^\text {Conditional total effect}= \end{aligned}$$

5 $$\begin{aligned}&\overbrace{g\{E[Y(a, M_{1}(a),M_{2}(a,M_{1}(a)))\vert C=c]\}-g\{E[Y(a, M_{1}(a^{*}),M_{2}(a^{*},M_{1}(a^{*})))\vert C=c]\}}^\text {Conditional natural indirect effect}+ \end{aligned}$$

6 $$\begin{aligned}&\overbrace{g\{E[Y(a, M_{1}(a^{*}),M_{2}(a^{*},M_{1}(a^{*})))\vert C=c]\}-g\{E[Y(a^{*}, M_{1}(a^{*}),M_{2}(a^{*},M_{1}(a^{*})))\vert C=c]\}}^\text {Conditional natural direct effect} \end{aligned}$$

Conditional and marginal effects have a similar interpretation, with the difference that the marginal effect is the average effect in the study sample and subsequently in the source population provided that the sample is representative of the population.

In the case-study, the conditional effects have the same definition of the marginal effects but conditionally on maternal age, education, residence and body mass index at the beginning of pregnancy, parity and child’s sex.

### Assumptions

The sufficient assumptions to identify the effects in the two-way decomposition reported above include the following [1, 4, 25], which are defined in terms of counterfactuals in the Supplemental Material:

- consistency: the counterfactuals $M_{1}(a), M_{2}(a,m_{1})$ and $Y(a,m_{1},m_{2})$ are equal to the observed $M_{1},M_{2}$ and *Y* when $A=a, M_{1}=m_{1}$ and $M_{2}=m_{2}$. Note that when exposures and mediators, alone or in combination, have drastically different effects on the potential outcomes in sub-groups, or analytically when effects heterogeneity is not modelled, the consistency assumption is likely to be violated [26];
- positivity: there are no empty cells or zero values either biologically or by design for the probabilities of $M_{2}$ given $M_{1},A$ and *C*, of $M_{1}$ given *A* and *C*, of *A* given *C*;
- no unmeasured and/or uncontrolled confounding of the exposure-outcome association, mediators-outcome association and exposure-mediators association;
- cross-world independence assumption: it states that there is independence between the counterfactual outcome and mediators values across worlds with one being a world in which the exposure is set to $A=a$ for the outcome and the other being a world in which the exposure is set to $A=a^{*}$ for the mediators. It assumes the absence of i)confounding of the effects of the mediators $M_{1}$ and $M_{2}$ on the outcome *Y* affected by the exposure A [27] (i.e. no measured or unmeasured intermediate confounders) and ii)latent variables acting as confounders across the different interventional settings of the exposure A (i.e. a latent variable U that affects specifically $M_{1}(a^{*}), M_{2}(a^{*},m_{1})$ and $Y(a,m_{1},m_{2})$) [28]. Note that in presence of intermediate confounding an option is to consider the intermediate confounder as an additional mediator and consider all mediators in the analysis extending the cross-world independence to the additional mediator. Alternatively sensitivity analyses can be carried out to explore the possible impact of the cross-world independence violation [28–31].

### Selected methods for multiple mediation analysis

In this section, when describing the implementation of the four selected methods, we consider that the exposure A, the mediators $M_{1}$ and $M_{2}$, and the outcome Y are all binary. However, these methods can be implemented in scenarios with different combinations of continuous, categorical, count and binary variables, as specified below.

The main characteristics of each approach are summarised in Table 1. The derivation of the effects and the procedure to apply each described method in practice are described in details in the Supplemental material.

**Table 1:** Main characteristics of each of four approaches

Decomposition of total effect	IORW$$^{*}$$	IPW$$^{**}$$	Imputation	Extended imputation
Two-way	$\checkmark$	$\checkmark$	$\checkmark$	$\checkmark$
Three-way				$\checkmark$
Type of estimated effects				
Marginal		$\checkmark$	$\checkmark$	$\checkmark$
Conditional	$\checkmark$		$\checkmark$	$\checkmark$
Models for				
Outcome	$\checkmark$	$\checkmark$	$\checkmark$	$\checkmark$
Mediators$^{\#}$				$\checkmark$
Exposure	$\checkmark$	$\checkmark$		
Nested counterfactual			$\checkmark$	$\checkmark$
Exposure type				
Binary	$\checkmark$	$\checkmark$	$\checkmark$	$\checkmark$
Categorical	$\checkmark$	$\checkmark$	$\checkmark$	$\checkmark$
Count				
Continuous	$\surd ^{++}$	$\surd ^{++}$	$\checkmark$	$\checkmark$
Outcome type				
Binary	$\checkmark$	$\checkmark$	$\checkmark$	$\checkmark$
Categorical	$\checkmark$	$\checkmark$	$\checkmark$	$\checkmark$
Count	$\checkmark$	$\checkmark$	$\checkmark$	$\checkmark$
Continuous	$\checkmark$	$\checkmark$	$\checkmark$	$\checkmark$
Mediator type				
Binary	$\checkmark$	$\checkmark$	$\checkmark$	$\checkmark$
Categorical	$\checkmark$	$\checkmark$	$\checkmark$	$\checkmark$
Count	$\checkmark$	$\checkmark$	$\checkmark$	$\checkmark$
Continuous	$\checkmark$	$\checkmark$	$\checkmark$	$\checkmark$
Interactions				
Exposure-mediators	$^{\#\#}$	$\checkmark$	$\checkmark$	$\checkmark$
Exposure-covariates	$\checkmark$	$\checkmark$	$\checkmark$	$\checkmark$
Mediator-mediator	$\checkmark$	$\checkmark$	$\checkmark$	$\checkmark$
Mediators-covariates	$\checkmark$	$\checkmark$	$\checkmark$	$\checkmark$

^*^Inverse odds ratio weighting.

^**^ Inverse probability weighting.

^#^ The presented methods circumvent the difficulty of specifying a regression model for the joint density of multiple mediators, with the exception of the extended imputation approach, which requires the specification of a model for the first or the second mediator in presence of two mediators.

^++^ The performance improves as the exposure is binary or categorical with few levels.

^##^ IOR is equally valid regardless of whether such interactions are present, without having to specify them, since the mediators are never entered into the regression model for the outcome and are only used to calculate the weights which are obtained by a regression model of the exposure on the mediators and the covariates

The *inverse odds ratio weighting approach* [13, 14] (IOR) is based on the calculation of the inverse odds ratio weights, which are used to make the exposure and the mediators independent and hence deactivate the indirect pathways of the mediators.

1. It estimates the conditional direct and indirect effects within the levels of the covariates *C* (expressions (4), (5), (6)). Based on the odds ratio’s invariance property, weights are calculated by regressing the exposure on the mediators and the covariates. The exposure’s coefficient in the weighted regression model for the outcome estimates the natural direct effect of the exposure on the outcome and the natural indirect effect is calculated as difference/ratio between the total effect and the direct effect. All the conditional effects are estimated using the original data with no imputations.
2. The implementation of this approach does not require models for the mediators while it requires to specify a regression model for the exposure given the mediators and the covariates to calculate the weights, and a weighted regression model for the outcome given the exposure and the covariates.
3. Interactions between the mediators can be included in the regression model for the exposure but exposure-mediator interactions do not need to be specified since the mediators are never entered into the regression model for the outcome.

The *inverse probability weighting approach* (IPW) [12] is based on the calculation of the inverse probability weights, which are used to make the exposure and the covariates independent.

1. It estimates the marginal natural direct and natural indirect effects (expressions (1), (2) and (3)) through the estimate of the three counterfactuals, $g\{E[Y(a, M_{1}(a),M_{2}(a,M_{1}(a)))]\}$, $g\{E[Y(a^{*}, M_{1}(a^{*}),M_{2}(a^{*},M_{1}(a^{*})))]\}$ and $g\{E[Y(a, M_{1}(a^{*}),M_{2}(a^{*},M_{1}(a^{*})))]\}$. The first two counterfactuals can be estimated from the observed data. The third counterfactual $g\Big \{E[Y(a, M_{1}(a^{*}),M_{2}(a^{*},M_{1}(a^{*})))]\Big \}$, which includes potential outcomes under both $A=a$ and $A=a^{*}$, cannot be obtained by the observed data but can still be estimated by standardising the mean outcome *Y* in each stratum defined by the mediators $M_{1}$ and $M_{2}$ and the confounders *C* among individuals exposed at the level $A=a$, to the mediator distribution of individuals exposed at the level $A=a^{*}$ and by weighting by the reciprocal of the conditional probability of the exposure A given the covariates C. This is an imputation procedure where the observed data are complemented with imputed data in which the same individual is evaluated at different exposure levels, *a* and $a^{*}$, but corresponding to the observed mediator levels and confounders. Applying inverse probability weighting entails calculating a weighted average of the imputed counterfactual outcomes to obtain marginal estimates of the effects.
2. The implementation of this approach does not require models for the mediators while it requires to specify a regression model for the exposure conditional on confounders and for the outcome conditional on the exposure, the mediators, and the confounders.
3. Exposure-mediator interactions and interactions between mediators can be included in the regression model for the outcome.

The *imputation approach* [15] is based on the so-called natural effects models, i.e. structural models for nested counterfactuals that directly parameterise the natural direct and indirect effects [32].

1. It estimates both the marginal and conditional natural direct and indirect effects (expressions (1), (2), (3), (4), (5), (6)). We introduce it here focusing on the conditional effects. The natural effects models express the nested counterfactual $g\{E[Y(a^{\prime \prime }, M_{1}(a^{\prime }),M_{2}(a^{\prime },M_{1}(a^{\prime })))\vert C=c]\}$ in terms of two newly defined ’“exposure” variables $A^{\prime }$ and $A^{\prime \prime }$ assuming the same potential levels of *A* (if *A* is binary with two levels 0 and 1, then $A^{\prime }$ and $A^{\prime \prime }$ have also two hypothetical levels 0 and 1). Their inclusion in the regression model allows to encode two causal pathways: through neither mediator (i.e direct pathway $A\rightarrow Y$), or through at least one of the two mediators (i.e. indirect pathways $A\rightarrow M_{1}\rightarrow Y, A\rightarrow M_{1}\rightarrow M_{2}\rightarrow Y, A\rightarrow M_{2}\rightarrow Y$, for brevity: $A\rightarrow M_{1}M_{2}Y)$. Similarly to the IPW approach, the nested counterfactual $g\{E[Y(a^{\prime \prime }, M_{1}(a^{\prime }),M_{2}(a^{\prime },M_{1}(a^{\prime })))\vert C=c]\}$ can be estimated from the observed data when $a^{\prime \prime }$ and $a^{\prime }$ equal the observed exposure *A* ($a^{\prime \prime }$ corresponds to *a* and $a^{\prime }$ to $a^{*}$ in the IPW) . When $a^{\prime }$ is equal to the observed exposure *A*, while $a^{\prime \prime }$ differs from $a^{\prime }$ then $g\{E[Y(a^{\prime \prime }, M_{1}(a^{\prime }),M_{2}(a^{\prime },M_{1}(a^{\prime })))\vert C=c]\}$ can still be estimated by standardising the mean outcome *Y* in each stratum defined by the mediators $M_{1},M_{2}$ and the confounders *C* among individuals exposed at the level $A=a^{\prime \prime }$, to the mediator distribution of individuals exposed at the level $A=a^{\prime }$. This gives arise to an imputation procedure where the observed data are complemented with imputed data in which the same individual is evaluated at different exposure levels, $a^{\prime }$ and $a^{\prime \prime }$, but corresponding to the observed mediator levels and confounders. The imputed data are then regressed on $A^{\prime }$, $A^{\prime \prime }$ and *C* and the conditional natural direct and indirect effects are obtained through linear combinations of the estimated parameters. The estimation of the marginal effects can be performed by weighting the natural effects model $g\{E[Y(a^{\prime \prime }, M_{1}(a^{\prime }),M_{2}(a^{\prime },M_{1}(a^{\prime })))]\}$ by the reciprocal of the conditional probability of the exposure A given the covariates C estimated using a logistic regression. The imputation approach differs from the IPW approach in the estimation of the effects: the former uses the natural effects model, while the latter calculates a weighted average of the imputed counterfactual outcomes.
2. The implementation of this approach does not require models for the mediators (averaging is performed over the empirical distribution of the joint mediators), while it requires to specify a regression model for the outcome conditional on the exposure, the mediators, and the confounders (imputation model), and a regression model for the nested counterfactual (natural effects model).
3. Exposure-mediator interactions and interactions between the mediators can be included in the regression model for the outcome and consequently in the natural effects model (by including the interaction between $a^{\prime \prime }$ and $a^{\prime }$).

Note that for all the approaches considered so far, as $M_{1}$ and $M_{2}$ are sequential, one could first consider $M_{1}$ alone and estimate the portion of the effect mediated through $M_{1}$ and then consider $M_{1}$ and $M_{2}$ jointly and estimate the portion of the effect mediated through $M_{1}$ and $M_{2}$. As $M_{1}$ and $M_{2}$ share a common pathway (i.e. the path going from *A* to $M_{1}$ and then to $M_{2}$ and *Y* jointly), the difference between the effects estimated by these two analyses may be different from the portion of the effect mediated through $M_{2}$ alone. However, it can be of interest to evaluate the additional contribution of $M_{2}$ beyond $M_{1}$ alone, and hence to decompose the indirect effect into the effect mediated through $M_{1}$ (i.e. the two pathways $A\rightarrow M_{1}\rightarrow Y, A\rightarrow M_{1}\rightarrow M_{2}\rightarrow Y$) and the effect mediated through $M_{2}$ alone (i.e. the pathway $A\rightarrow M_{2} \rightarrow Y)$ as follows:

7 $$\begin{aligned}&\overbrace{g\{E[Y(a, M_{1}(a),M_{2}(a,M_{1}(a)))\vert C=c]\}-g\{E[Y(a, M_{1}(a^{*}),M_{2}(a^{*},M_{1}(a^{*})))\vert C=c]\}}^\text {Conditional indirect effect}= \end{aligned}$$

8 $$\begin{aligned}&\overbrace{g\{E[Y(a, M_{1}(a),M_{2}(a,M_{1}(a)))\vert C=c]\}-g\{E[Y(a, M_{1}(a^{*}),M_{2}(a,M_{1}(a^{*})))\vert C=c]\}}^{\text {Conditional indirect effect through }M_{1}}+ \end{aligned}$$

9 $$\begin{aligned}&\overbrace{g\{E[Y(a, M_{1}(a^{*}),M_{2}(a,M_{1}(a^{*})))\vert C=c]\}-g\{E[Y(a, M_{1}(a^{*}),M_{2}(a^{*},M_{1}(a^{*})))\vert C=c]\}}^{\text {Conditional partial indirect effect through } M_{2}\;\text {alone }} \end{aligned}$$

The indirect effect through $M_{1}$ captures all pathways along $M_{1}$ to *Y* further mediated or not mediated by $M_{2}$ ($A\rightarrow M_{1}\rightarrow Y, A\rightarrow M_{1}\rightarrow M_{2}\rightarrow Y$). The partial indirect effect through $M_{2}$ captures all pathways along $M_{2}$ to *Y* not passing through $M_{1}$ ($A\rightarrow M_{2}\rightarrow Y$). In order to estimate these effects, the two usual additional assumptions need to be satisfied, namely the absence of unmeasured confounding of the $M_{1}-M_{2}$ association and the lack of confounders of this association in turn affected by the exposure.

The *extended imputation approach* [16], being an extension of the imputation approach to further decompose the natural indirect effect into the effect mediated through $M_{1}$ and the effect mediated through $M_{2}$ alone, is also based on the natural effects model.

1. It estimates both the marginal and conditional direct and indirect effects through $M_{1}$ and through $M_{2}$ alone. Considering conditional effects, the nested counterfactual $g\{E[Y(a^{\prime \prime \prime }, M_{1}(a^{\prime }),M_{2}(a^{\prime \prime },M_{1}(a^{\prime })))\vert C=c]\}$ is now defined in terms of three newly defined “exposure” variables $A^{\prime }$, $A^{\prime \prime }$ and $A^{\prime \prime \prime }$. $A^{\prime }$, $A^{\prime \prime }$ and $A^{\prime \prime \prime }$ are three variables with the same potential levels of *A* (if *A* is binary with two levels 0 and 1, then $A^{\prime }$, $A^{\prime \prime }$ and $A^{\prime \prime \prime }$ have also two hypothetical levels 0 and 1), and their inclusion in the regression model allows to encode the three causal pathways of interest, through neither of the mediators (i.e. the direct pathway $A\rightarrow Y$), through $M_{1}$ or $M_{1}$ and then $M_{2}$ (i.e. the indirect pathway through $M_{1}$: $A\rightarrow M_{1}\rightarrow Y, A\rightarrow M_{1}\rightarrow M_{2}\rightarrow Y$) or through $M_{2}$ alone (i.e. the partial indirect pathway through $M_{2}$: $A\rightarrow M_{2}\rightarrow Y$). The nested counterfactual $g\{E[Y(a^{\prime \prime \prime }, M_{1}(a^{\prime }),M_{2}(a^{\prime \prime },M_{1}(a^{\prime })))\vert C=c]\}$ can be estimated from the observed data when $a^{\prime \prime \prime }$, $a^{\prime \prime }$ and $a^{\prime }$ equal the observed exposure *A*. When $a^{\prime \prime \prime }$, $a^{\prime \prime }$ and $a^{\prime }$ differ one from the others, $g\{E[Y(a^{\prime \prime \prime }, M_{1}(a^{\prime }),M_{2}(a^{\prime \prime },M_{1}(a^{\prime })))\vert C=c]\}$ can still be estimated by (i) standardising the mean outcome *Y* in each stratum defined by the mediators $M_{1},M_{2}$ and the confounders *C* among individuals exposed at the level $A=a^{\prime \prime \prime }$, to the mediator distribution of individuals exposed at the level $A=a^{\prime \prime }$ and by weighting for a weight that models the probability of $M_{1}$ under different scenarios of the exposure ($A=a^{\prime }$ or $A=a^{\prime \prime }$), or (ii) standardising the mean outcome *Y* in each stratum defined by the mediators $M_{1},M_{2}$ and the confounders *C* among individuals exposed at the level $A=a^{\prime \prime \prime }$, to the mediator distribution of individuals exposed at the level $A=a^{\prime }$ and by weighting for a weight that models the probability of $M_{2}$ under different scenarios of the exposures ($A=a^{\prime }$ or $A=a^{\prime \prime }$). The estimation of the marginal effects can be performed by weighting the natural effects model $g\{E[Y(a^{\prime \prime \prime }, M_{1}(a^{\prime }),M_{2}(a^{\prime \prime },M_{1}(a^{\prime })))]\}$ by the reciprocal of the conditional probability of the exposure A given the covariates C estimated using a logistic regression.
2. The implementation of this approach does require to specify a regression model for one of the two mediators, a regression model for the outcome conditional on the exposure, the mediators, and the confounders (imputation model), and a regression model for the nested counterfactual (natural effects model). According to the confidence on the model’s correct specification, one can choose as where to model the distribution of the first or the second mediator.
3. Exposure-mediator interactions and interactions between the mediators can be included in the regression model for the outcome and consequently in the natural effects model (by including the interaction between $A=a^{\prime \prime \prime }$,$A=a^{\prime \prime }$ and $A=a^{\prime }$).

## Results

Out of 4797 mother-child pairs, 7% of mothers had depression or anxiety during pregnancy. The prevalence of adverse reproductive outcomes, as defined above, was 31% and the prevalence of lower respiratory infections in the first 6 months of infant life was 11%. The prevalence of wheezing between 6 and 18 months of infant life was 17%. Some 26% of the infants born to mothers affected by depression or anxiety during pregnancy had wheezing between 6 and 18 months of life *vs* 16% of those born to mothers without depression or anxiety during pregnancy. We use a Poisson regression to model risk ratio and prevalence ratio. We find a 37% increased prevalence of adverse reproductive outcomes in women with depression or anxiety in pregnancy compared to those without these conditions (PR: prevalence ratio, PR adjusted for *C*: 1.37, CI: confidence interval, 95% CI: 1.20;1.55), a 29% increased risk of lower respiratory infection in the first 6 months (RR: risk ratio, RR adjusted for *C*: 1.29, 95% CI: 0.95;1.76) and a 64% increased prevalence of wheezing (PR adjusted for *C*: 1.64, 95% CI: 1.35: 1.99). Adverse reproductive outcomes are associated with a 19% increased risk of lower respiratory infections in the first 6 months (RR adjusted for *A* and *C*: RR=1.19, 95% CI: 0.98; 1.43) and a 23% increased prevalence of infant wheezing (PR adjusted for *A* and *C*: PR=1.23, 95% CI: 1.06;1.40). Finally lower respiratory infections in the first 6 months double the prevalence of infant wheezing between 6 and 18 months of life (PR adjusted for *A*, $M_{1}$ and *C*: PR=2.03, 95% CI: 1.75;2.35).

A summary of the fitted regression models with their R code to implement each of the four approaches to sequential mediation analysis is reported in the Supplemental Material.

Results of the sequential analyses performed using the inverse odds ratio weighting, the inverse probability weighting and the imputation approaches are reported in Table 2, while results obtained using the extended imputation approach are reported in Table 3.

**Table 2:** Estimates of total, direct and indirect effects of maternal depression or anxiety in pregnancy on the risk of infant wheezing between 6 and 18 months of age from inverse odds ratio weighting, inverse probability weighting and imputation approach. $M_{1}$: adverse reproductive outcomes. $M_{2}$: infant lower respiratory infections

	Through $M_{1}$		Through $M_{1}$ and $M_{2}$	
	PR	95% CI*	PR	95% CI
Conditional effect	IOR$^{*}$ approach			
Direct effect	1.59	1.27-1.94	1.57	1.25-1.92
Indirect effect	1.03	0.94-1.12	1.05	0.95-1.15
Total effect	1.64	1.33-2.00	1.64	1.33-1.97
Marginal effect	IPW$$^{**}$$ approach			
Direct effect	1.60	1.30-1.94	1.57	1.27-1.87
Indirect effect	1.02	0.99-1.04	1.04	0.99-1.09
Total effect	1.63	1.33-1.98	1.63	1.31-1.95
Conditional effect	Imputation approach			
Direct effect	1.60	1.31-1.94	1.57	1.26-1.90
Indirect effect	1.02	1.01-1.05	1.05	1.01-1.09
Total effect	1.64	1.33-1.99	1.64	1.33-1.99
Marginal effect	Imputation approach			
Direct effect	1.60	1.30-1.91	1.57	1.24-1.88
Indirect effect	1.02	1.00-1.04	1.04	0.99-1.09
Total effect	1.63	1.33-1.95	1.62	1.29-1.95

PR: prevalence ratio; CI: confidence interval calculated by bootstrap.

^*^ Inverse odds ratio weighting.

^**^ Inverse probability weighting

**Table 3:** Estimates of conditional total, direct and indirect effects by extended imputation approach. $M_{1}$: adverse reproductive outcomes. $M_{2}$: infant lower respiratory infections

	Extended imputation approach	
Conditional effect	PR	95% CI
Direct effect	1.57	1.28-1.86
Indirect effect through $M_{1}$ and $M_{2}$ jointly	1.05	1.00-1.09
Indirect effect through $M_{1}$	1.00	0.99-1.00
Partial indirect effect through $M_{2}$	1.05	1.00-1.09
Total effect	1.64	1.34-1.96

PR: prevalence ratio; CI: confidence interval calculated by bootstrap

The *inverse odds ratio weighting approach* suggests that being born to a mother with depression or anxiety in pregnancy compared to a mother not suffering from these disorders increases the prevalence of infant wheezing (PR=1.64, 95% CI: 1.33-2.00). Being born to a mother with depression or anxiety in pregnancy compared to a mother not suffering from these conditions, while setting presence of adverse reproductive outcomes as naturally observed in the absence of maternal depression or anxiety in pregnancy, increases the prevalence of infant wheezing (natural direct effect when only $M_{1}$ is considered: PR=1.59, 95% CI: 1.27-1.94). Comparing levels of adverse reproductive outcomes that would have been observed in presence of maternal depression or anxiety in pregnancy to levels that would been observed in absence of maternal depression or anxiety in pregnancy, while setting maternal depression or anxiety in pregnancy as present, increases only minimally the prevalence of infant wheezing (natural indirect effect when only $M_{1}$ is considered PR=1.03, 95% CI: 0.94, 1.12). Similarly being born to a mother with depression or anxiety in pregnancy compared to a mother not suffering from these disorders, while setting the presence of adverse reproductive outcomes and lower respiratory infections as naturally observed in absence of maternal depression or anxiety in pregnancy, increases the prevalence of infant wheezing (natural direct effect when $M_{1}$ and $M_{2}$ are considered jointly: PR=1.57, 95% CI: 1.25-1.92). Comparing levels of adverse reproductive outcomes and lower respiratory infections that would have been observed in presence of maternal depression or anxiety in pregnancy to levels that would been observed in absence of maternal depression or anxiety in pregnancy, while setting the maternal depression or anxiety in pregnancy as present, increases only minimally the prevalence of infant wheezing (natural indirect effect when $M_{1}$ and $M_{2}$ are considered jointly: PR=1.05, 95% CI: 0.95, 1.15).

In summary, the direct effect of maternal depression or anxiety in pregnancy is equal to a 59% (95% CI: 27%-94%) increased prevalence of infant wheezing and the mediated effect through adverse reproductive outcomes is equal to a 3% (95% CI: -6%-12%) increased prevalence of infant wheezing. When including infant lower respiratory infections in the mediation pathway, the direct effect decreases slightly to 57% (95% CI: 25%-92%) and consequently the indirect effect increases slightly to 5% (95% CI: -5%,15%). Hence although adverse reproductive outcomes and infant lower respiratory infections are both risk factors for infant wheezing and are affected by maternal depression or anxiety in pregnancy, they explain only minimally the observed increased risk of infant wheezing associated with maternal depression or anxiety in pregnancy [33–35]. This exposure acts on infant wheezing through other mechanisms/pathways that are not considered in our case-study analysis.

The corresponding estimates of the natural direct and indirect effects obtained using the *weighting approach* and the *imputation approach* are very similar to those described above, although the inverse odds ratio weighting approach has slightly larger confidence intervals for the direct and indirect effects. The *extended imputation approach* suggests further that the small joint indirect effect through adverse reproductive outcomes and lower respiratory infections is due entirely to the contribution of infant lower respiratory infections (PR=1.05, 95% CI: 1.00,1.09), independently from the increased prevalence of adverse reproductive outcomes.

Note that the approaches described in this paper assume that the conditional effects are the same for any level of baseline confounders unless in presence of exposure-confounder interaction. Hence, also conditional and marginal effects are expected not to differ because interactions between the exposure and the baseline covariates are not included in the regression models. In our case-study we considered the interaction between the two mediators, while we assumed absence of the interaction between the exposure and the baseline covariates and the three-way interaction between the exposure and the mediators. However, all methods can further consider these interactions with the exclusion of the inverse odds ratio weighting approach that cannot specify the three-way interaction. When we included the latter in the analysis, similar results of lack of indirect effects were obtained.

Note that the estimated effects can be considered as causal only if the assumptions specified above hold.

## Discussion

In this paper we applied to case-study of interest for birth cohort research four different estimation approaches recently developed to answer research questions involving sequential mediation analysis. We described the methods in details and provided the code to stimulate the implementation of these approaches in future studies. The four methods revealed similar results of small mediating role of adverse reproductive outcomes and early respiratory tract infections in the effect of maternal pregnancy mental health on infant wheezing.

The interest in using these methods can be twofolds: on the one hand they allow the study of multiple mechanisms underlying the association between an exposure and an outcome, on the other they provide a possible solution for the problem of intermediate confounding by considering the intermediate confounder as a sequential mediator in the analysis. However, the correct estimation of natural direct and indirect effects relies on several assumptions (on the top of the issue of intermediate confounder): the absence of unmeasured confounders of the exposure-outcome, exposure-mediators, mediators-outcome associations in all four approaches, the absence of unmeasured confounders of the association between the sequential mediators and the absence of the confounders of this association if affected by the exposure in the extended imputation approach, the correct specification of the models for i)the outcome in all four approaches, ii)the exposure in the inverse odds ratio and inverse probability weighting approaches, iii)at least one mediator in the extended imputation approach, and iv)the nested counterfactual in the imputation and the extended imputation approaches. The choice of the method may depend on the nature of the variables involved in the analysis and the user’s prior modelling knowledge and confidence in the underlying assumptions: for example, the inverse odds ratio and the inverse probability weighting could be preferred when the mediators are more difficult to model than exposure (e.g. continuous mediators and binary exposure), while the imputation approaches may be the first option when it is more difficult to specify the model for the exposure than for mediators (e.g continuous exposure and binary mediators). It is also important to consider what is the effect of main interest: the inverse odds ratio approach estimates the conditional direct and indirect effects, the inverse probability weighting estimates the marginal direct and indirect effects, while the imputation and the extended imputation approaches can estimate both conditional and marginal direct and indirect effects. Finally, the extended imputation approach is the only method that allows the decomposition of the natural indirect effect into the effect mediated through the first mediator and the effect mediated through the second mediator alone. This further decomposition of the total effect occurs at a price as it requires the specification of an additional model for one of the two mediators contrarily to other approaches which do not require any model for the mediators.

In the case-study, to estimate the conditional effects, we modelled the outcome by a Poisson regression model conditional on the exposure, mediators and covariates in the IPW, the imputation approach and the extended imputation approach and conditional on the exposure and covariates in the IOR approach. We modelled the exposure by a logistic regression model conditional on mediators and covariates in the IOR approach and conditional on the covariates in the IPW approach. We modelled the nested counterfactuals by a Poisson regression model conditional on the newly created variables for the exposure and the covariates in the imputation approach and its extension. Finally we modelled the first mediator by a Poisson regression model conditional on the newly created variables for the exposure and the covariates and the second mediator by a Poisson regression model conditional on the first mediator, the newly created variables for the exposure and the covariates in the extended imputation approach. Despite these differences in the models’ specification, the four estimation methods led to similar conclusions in our case-study, namely that the effect of maternal mental health on infant wheezing is not mediated by adverse reproductive outcomes and infant lower respiratory infections. This is reassuring for what regards the underlying assumptions used in mediation analysis, in particular the assumption on the correct specification of the model.

In this article we focused on the application of the methods to the context with two sequential mediators. In presence of multiple mediators, one could for simplicity consider a group of mediators as a joint mediator as we did for adverse reproductive outcomes. Alternatively the approaches can be extended to settings with more than two mediators with caution in underlying identification and estimation assumptions and modelling. Steen at al (2017) [16] showed how to fit the extended imputation approach to these contexts. Some other methods provide a finer decomposition of the total effect than the methods addressed in this paper, yet they may relay on stronger assumptions [2, 19]. As opposed to Monte Carlo approaches, which require to model the joint density of the mediators, the extended imputation approach requires to model the density of only one of the mediators in presence of two mediators. However, when the joint density is correctly specified, fully parametric Monte Carlo approaches yield more efficient estimators for specific direct and indirect effects along each of the possible mediation pathways. A comparison between the extended imputation approach [16] and the Monte Carlo estimation procedure proposed by Daniel et al (2015) [2] was carried out by Ananth and Loh (2022) [36] and the different procedures yielded very similar results on the estimated effects in common.

For the sake of completeness, it is worth mentioning here an approach that was not applied in this tutorial. Vansteenlandt and Daniel (2017) [37] revisited and refined the interventional direct and indirect effects [38] in presence of multiple mediators, and showed how the total effect can be decomposed into these effects. Briefly, the interventional effects differ from the natural effects because, instead of setting the mediator to the counterfactual level it would have naturally taken under different scenarios of the exposure, it sets the mediator for each subject to a random draw from the counterfactual distribution of mediator given the covariates under different scenarios of the exposure. The interventional effects are particularly relevant about a policy that involves fixing the mediator distribution, or shifting it to the extent that it is affected by the exposure, and they can be identified under weaker conditions than natural effects, i.e. exposure-induced confounder of the mediator-outcome association can be present and the cross-world independence assumption is not required. Loh et al (2020) [39] generalized natural effect models and the (extended) imputation approach to estimate conditional interventional effects for multiple mediators.

In the sequential mediation analysis there are still unsolved methodological issues, which, although of interest, go beyond the scope of this work, for example the degree of bias in the estimates when the underlying assumptions of each approach are violated or when the variables involved in the mediation pathways are poorly measured.

## Conclusions

As the need to use sequential mediation analysis is becoming increasingly common in epidemiology and the proposed methods are not easy to implement, the aim of this work is to help applied epidemiologists to run valid sequential mediation analysis whenever required by their research hypothesis. It provides a detailed overview and step-by-step implementation with the statistical software R of four weight-based and/or imputation-based methods to analyse multiple sequential mediators in a causal inference framework using a case-study of interest for birth cohort research.

## Supplementary Information

**Additional file 1.**

## Data Availability

The datasets used and/or analysed during the current study are available from the corresponding author on reasonable request. The code implemented in R is available in the Supplemental Material.

## Abbreviations

IOR: Inverse odds ratio weighting approach
IPW: Inverse probability weighting approach
PR: Prevalence ratio
CI: Confidence interval
