# Supplementary material for: Applied causal inference methods for sequential mediators
Source: BMC Med Res Methodol. 2022 Nov 24;22:301. doi: 10.1186/s12874-022-01764-w (PMC9686042; doi:10.1186/s12874-022-01764-w)
Supplement: Supplementary file 1 — Additional file 1. [file 12874_2022_1764_MOESM1_ESM.pdf]

Supplemental material

Description of the variables involved in the sequential mediation analysis

The aim of the case-study was to investigate the role of some suggested mechanisms underlying the effect of maternal mental health during pregnancy on infant wheezing between 6 and 18 months. Maternal mental health during pregnancy includes maternal depression and anxiety, defined as maternally reported doctor-diagnosed depression and/or anxiety active during the index pregnancy. The first mediator, adverse reproductive outcomes, includes any of the following: i) low birth weight (<2500 grams at birth), ii) preterm birth (<37 gestational weeks ta birth), and iii) cesarean delivery. The second mediator, lower respiratory tract infections, includes at least one episode of bronchitis, bronchiolitis or laryngitis in the first six months of life. The outcome, infant wheezing, is defined at at least one episode of wheezing or whistling in the chest occurring between 6 and 18 months of age. Maternal age, education, residence, and body mass index at the beginning of pregnancy, parity and child's sex are considered as baseline confounders. The maternal education is categorised in low-primary education or less, medium-secondary school, and high-university or higher, the maternal residence is categorised in north, center and south of Italy, body mass index at the beginning of pregnancy is categorised in underweight <18.5 kg/m2, normal weight 18.5-25.0 kg/m2, overweight 25-30 kg/m2, and obesity $\geq$ 30 kg/m2, the parity is categorised in nulliparous and more than one previous pregnancies.

Identifying assumptions for the direct and indirect effects

We use the notation $A \coprod \{B|C\}$ to denote that A is independent of B conditional on C, and $P(B|A, C)$ to denote the conditional probability of B given A and C. When B is continuous, the conditional probability is replaced by the conditional density. The identifying and estimating assumptions for the direct and indirect effects include:

- consistency:
  $P[M_1(a) = M_1|A = a] = 1$ for all $a \in A$,
  $P[M_2(a, m_1) = M_2|A = a, M_1 = m_1] = 1$ for all $a \in A$ and $m_1 \in M_1$,
  $P[Y(a, m_1, m_2) = Y|A = a, M_1 = m_1, M_2 = m_2] = 1$ for all $a \in A, m_1 \in M_1$ and $m_2 \in M_2$,

- positivity:
  $P(M_1 = m_1|A = a, C = c) > 0$ for all $m_1 \in M_1$,
  $P(M_2 = m_2|M_1 = m_1, A = a, C = c) > 0$ for all $m_2 \in M_2$,
  $P(A = a|C = c) > 0$ for all $a \in A$,

- no unmeasured confounding of the following effects:
  the exposure A on the outcome Y conditional on C $(Y(am_1m_2) \coprod A|C = c)$,
  both mediators M_1 and M_2 on the outcome Y conditional on A and C $(Y(am_1m_2) \coprod \{M_1, M_2\}|A = a, C = c)$,
  the exposure A on both mediators M_1 and M_2 conditional on C $(\{M_1, M_2\} \coprod A|C = c)$,

- the cross-world independence assumption: $Y(am_1m_2) \coprod \{M_1(a^*), M_2(a^*)\}|C = c)$.

Selected methods for multiple mediation analysis

We will describe the following four methods: the inverse odds ratio weighting approach (IOR), the inverse probability weighting approach (IPW), the imputation approach and the extended imputation approach. Specifically we will consider that the exposure A, the mediators M_1 and M_2, and the outcome Y are all binary. However, these methods can be implemented in scenarios with different combinations of continuous, categorical, count and binary variables.

Inverse odds ratio weighting approach

The inverse odds ratio weighting approach estimates the counterfactual $g\{E[Y(a, M_1(a^*), M_2(a^*, M_1(a^*)))|C = c]\}$ (i.e the scenario under which the population is exposed to $A = a$ but all the mediators take the natural value under the scenario $A = a^*$) by means of the following equality:

$$g\Big\{E\Big[\frac{P(M_1 = m_{01}, M_2 = m_{02}|A = a, C = c)}{P(M_1 = m_{01}, M_2 = m_{02}|A = a^*, C = c)}Y(a, M_1(a^*), M_2(a^*, M_1(a^*)))|C = c\Big]\Big\} = g[E(WY|A = a, C = c)] \tag{1}$$

where W is the inverse of the conditional odds ratio function relating M_1 and M_2 to A within the levels of C:

$$W = \frac{P(M_1 = m_{01}, M_2 = m_{02}|A = a, C = c)P(M_1 = m_1, M_2 = m_2|A = a^*, C = c)}{P(M_1 = m_{01}, M_2 = m_{02}|A = a^*, C = c)P(M_1 = m_1, M_2 = m_2|A = a, C = c)} \tag{2}$$

$P(M_1 = m_1, M_2 = m_2|A = a, C = c)$ is the joint conditional probability of the two mediators conditional on the exposure and the covariates, and m_{01} and m_{02} are the reference values of the two mediators.
Because of the invariance property of the odds ratio, the weight W can be also expressed as follows:

$$W = \frac{P(A = a^*|M_1 = m_1, M_2 = m_2, C = c)P(A = a|M_1 = m_{01}, M_2 = m_{02}, C = c)}{P(A = a|M_1 = m_1, M_2 = m_2, C = c)P(A = a^*|M_1 = m_{01}, M_2 = m_{02}, C = c)} \tag{3}$$

where $P(A = a|M_1 = m_1, M_2 = m_2, C = c)$ is the conditional probability of the exposure given the two mediators and the covariates.
Weighting each subject with the inverse odds ratio function (3) relating M_1 and M_2 to A within the levels of C makes A and $\{M_1, M_2\}$ independent. To obtain more stabilised weights, one can multiply each individual's exposure-mediator odds ratio by the predicted odds of the exposure when the mediators are fixed at their reference value obtaining an inverse odds weight instead of inverse odds ratio weight.

In practice (when considering A, M_1, M_2, Y binary), to estimate the conditional total effect we model the mean observed outcome for each subject (Y) conditional on the observed exposure (A) and the covariates (C) using a generalized linear regression model. The conditional total effect is then equal to the exponentiated coefficient for the exposure A if the interactions between A and C are not included in the model. To estimate the conditional pure direct effect we model the mean observed outcome for each subject (Y) conditional on the observed exposure (A) and the covariates (C) using a weighted generalized linear regression model where weights W are equal to 1 for unexposed subjects and equal to the inverse odds ratio predicted by the logistic regression model of A given M_1 and M_2 and C for the exposed subjects. The conditional pure direct effect is then equal to the inverse of the exponentiated coefficient for the exposure A if the interactions between A and C are not included in the model. Finally to estimate the conditional total indirect effect we calculate the ratio between the estimated total effect and the estimated pure direct effect. Note that all the effects are estimated using the original data with no imputations (Table 1). Confidence intervals can be calculated by bootstrapping.

Table 1: Inverse odds ratio weighting approach: example based on two subjects, one exposed (i=1) and the other one unexposed (i=2). A: exposure of the i-subiect, M_1: first mediator of the i-subiect, M_2: second mediator of the i-subiect, Y: outcome of the i-subiect.

i	A	$M_1(a)$	$M_2(a, M_1(a))$	$Y(a, M_1(a), M_2(a, M_1(a)))$
1	1	$M_1(1)$	$M_2(1, M_1(1))$	$Y(1, M_1(1), M_2(1, M_1(1)))$
2	0	$M_1(0)$	$M_2(0, M_1(0))$	$Y(0, M_1(0), M_2(0, M_1(0)))$

Inverse probability weighting approach

The inverse probability weighting approach estimates the marginal pure direct and total indirect effects. Specifically it estimates the three counterfactuals, $g\{E[Y(a, M_1(a), M_2(a, M_1(a)))]\}$, $g\{E[Y(a^*, M_1(a^*), M_2(a^*, M_1(a^*)))]\}$ and $g\{E[Y(a, M_1(a^*), M_2(a^*, M_1(a^*)))]\}$ by means of the following equalities:

$$g\Big\{E[Y(a, M_1(a), M_2(a, M_1(a)))]\Big\} = E\Big\{\frac{P(A = a)}{P(A = a|C = c)} g[E(Y|A = a, M_1 = m_1, M_2 = m_2, C = c)]|A = a\Big\} \quad (4)$$

$$g\Big\{E[Y(a^*, M_1(a^*), M_2(a^*, M_1(a^*)))]\Big\} = E\Big\{\frac{P(A = a^*)}{P(A = a^*|C = c)} g[E(Y|A = a^*, M_1 = m_1, M_2 = m_2, C = c)]|A = a^*\Big\} \quad (5)$$

$$g\Big\{E[Y(a, M_1(a^*), M_2(a^*, M_1(a^*)))]\Big\} = E\Big\{\frac{P(A = a^*)}{P(A = a^*|C = c)} g[E(Y|A = a, M_1 = m_1, M_2 = m_2, C = c)]|A = a^*\Big\} \quad (6)$$

The two counterfactuals $g\{E[Y(a, M_1(a), M_2(a, M_1(a)))]\}$ and $g\{E[Y(a^*, M_1(a^*), M_2(a^*, M_1(a^*)))]\}$ can be estimated from the observed data. The third counterfactual $g\Big\{E[Y(a, M_1(a^*), M_2(a^*, M_1(a^*)))]\Big\}$, which includes both two potential outcomes under $A = a$ and $A = a^*$ and cannot be obtained by the observed data, can still be estimated by standardising the mean outcome Y in each stratum defined by the mediators M_1 and M_2 and the confounders C among individuals exposed at the level $A = a$, to the mediator distribution of individuals exposed at the level $A = a^*$ and by weighting by the reciprocal of the conditional probability of the exposure A given the covariates C.

In practice (when considering A, M_1, M_2, Y binary), we fit to the observed data i) an outcome model $g[E(Y|A = a, M_1 = m_1, M_2 = m_2, C = c)]$ conditional on the observed exposure A, the mediators M_1 and M_2, and covariates C using a generalized linear regression model and, ii) an exposure model $P(A|C = c)$ conditional on the observed covariates C using a logistic regression model to calculate the corresponding weights. We expand the observed data by repeating each observation in the original data set twice and we consider one additional variable $A^{'}$ which is equal to the observed exposure A for the first replication and equal to the opposite of the observed exposure for the second replication (Table 2). When $A^{'}$ is equal to the observed A, we estimate $g\{E[Y(1, M_1(1), M_2(1, M_1(1)))]\}$ and $g\{E[Y(0, M_1(0), M_2(0, M_1(0)))]\}$ from the outcome model by using the observed data. On the contrary, when $A^{'}$ is different from the observed A, we estimate $g\{E[Y(1, M_1(0), M_2(0, M_1(0)))]\}$ from the outcome model by using the individual's own values of mediators M_1 and M_2 and confounders C in the unexposed subjects (A=0), but using $A^{'} = 1$, the opposite of the observed exposure A. Hence we calculate a weighted average of these predicted values for subjects with $A = 0$. Similarly we predict $g\{E[Y(0, M_1(1), M_2(1, M_1(1)))]\}$ from the outcome model by using the individual's own values of mediators M_1 and M_2 and confounders C in the exposed subjects (A=1), but using $A^{'} = 0$, the opposite of the observed exposure A, and we calculate a weighted average of these predicted values for subjects with $A = 1$. Confidence intervals can be calculated by bootstrapping.

Table 2: Inverse probability weighting approach: example based on two subjects, one exposed (i=1) and the other unexposed (i=2). Bold quantities indicate the unobserved counterfactual values for each subject. A: exposure of the i-subiect, M_1: first mediator of the i-subiect, M_2: second mediator of the i-subiect, Y: outcome of the i-subiect.

i	A	$A^{'}$	$M_1(a)$	$M_2(a, M_1(a))$	$Y(a^{'}, M_1(a, M_2(a, M_1(a))$
1	1	1	$M_1(1)$	$M_2(1, M_1(1))$	$Y(1, M_1(1), M_2(1, M_1(1)))$
1	1	**0**	$M_1(1)$	$M_2(1, M_1(1))$	$\mathbf{Y(0, M_1(1), M_2(1, M_1(1)))}$
2	0	0	$M_1(0)$	$M_2(0, M_1(0))$	$Y(0, M_1(0), M_2(0, M_1(0)))$
2	0	**1**	$M_1(0)$	$M_2(0, M_1(0))$	$\mathbf{Y(1, M_1(0), M_2(0, M_1(0)))}$

Imputation approach
The imputation approach estimates both the marginal and conditional natural direct and indirect effects. We introduce it here focusing on the conditional effects. This approach is based on the so-called natural effects models, i.e. structural models for nested counterfactuals that directly parameterise the natural direct and indirect effects. The natural effects models express the nested counterfactual $g\{E[Y(a^{''}, M_1(a^{'}), M_2(a^{'}, M_1(a^{'})))|C = c]\}$ in terms of two newly defined '"exposure" variables $A^{'}$ and $A^{''}$ (defined below) to compare as follows:

$$g\{E[Y(a^{''}, M_1(a^{'}), M_2(a^{'}, M_1(a^{'})))|C = c]\} = \theta H(a^{''}, a^{'}, c) \tag{7}$$

where $H(a^{''}, a^{'}, c)$ is a vector depending on $A^{''} = a^{''}, A^{'} = a^{'}, C = c$ and θ is a vector of regression parameters to estimate. For example, $\theta H(a^{''}, a^{'}, c)$ could be $\theta_0 + \theta_1 a^{''} + \theta_2 a^{'} + \theta_3 a^{''} a^{'} + \theta_4 c$. $A^{'}$ and $A^{''}$ are two variables with the same potential levels of A, and their inclusion in the regression model allows to encode two causal pathways: through neither mediator (i.e direct pathways $A \to Y$), or through at least one of the two mediators (i.e. indirect pathways $A \to M_1 \to Y, A \to M_1 \to M_2 \to Y, A \to M_2 \to Y$, for brevity: $A \to M_1 M_2 Y$). Suppose A is binary with two levels 0 and 1, $A^{'}$ and $A^{''}$ are also binary and have two potential levels 0 and 1. If both $A^{'}$ and $A^{''}$ are set to 1, the equation (7) is equal to $g\{E[Y(1, M_1(1), M_2(1, M_1(1)))|C = c]\} = \theta_0 + \theta_1 + \theta_2 + \theta_3 + \theta_4 c$, while, if both $A^{'}$ and $A^{''}$ are set to 0, the equation (7) is equal to $g\{E[Y(0, M_1(0), M_2(0, M_1(0)))|C = c]\} = \theta_0 + \theta_4 c$. Hence the conditional total effect is equal to:

$$g\{E[Y(1, M_1(1), M_2(1, M_1(1)))|C = c]\} - g\{E[Y(0, M_1(0), M_2(0, M_1(0)))|C = c]\} = \theta_1 + \theta_2 + \theta_3. \tag{8}$$

To decompose the total effect, it is necessary to consider scenarios in which $A^{''}$ is set to a different value than $A^{'}$. The conditional pure direct effect is equal to:

$$g\{E[Y(1, M_1(0), M_2(0, M_1(0)))|C = c]\} - g\{E[Y(0, M_1(0), M_2(0, M_1(0)))|C = c]\} = \theta_1 \tag{9}$$

and the conditional total indirect effect is equal to:

$$g\{E[Y(1, M_1(1), M_2(1, M_1(1)))|C = c]\} - g\{E[Y(1, M_1(0), M_2(0, M_1(0)))|C = c]\} = \theta_2 + \theta_3 \tag{10}$$

Similarly to IPW approach, the nested counterfactual $g\{E[Y(a^{''}, M_1(a^{'}), M_2(a^{'}, M_1(a^{'})))|C = c]\}$ can be estimated from the observed data when $a^{''}$ and $a^{'}$ equal the observed exposure A ($a^{''}$ corresponds to a and $a^{'}$ to a^* in the IPW) . When $a^{'}$ is equal to the observed exposure A, while $a^{''}$ differs from $a^{'}$ then $g\{E[Y(a^{''}, M_1(a^{'}), M_2(a^{'}, M_1(a^{'})))|C = c]\}$ can still be estimated according to the following equality:

$$g\{E[Y(a^{''}, M_1(a^{'}), M_2(a^{'}, M_1(a^{'})))|C = c]\} = \sum_{m_1, m_2} g[E(Y|A = a^{''}, M_1 = m_1, M_2 = m_2, C = c)]P(M_1 = m_1, M_2 = m_2|A = a^{'}, C = c) \tag{11}$$

It consists of standardising the mean outcome Y in each stratum defined by the mediators M_1, M_2 and the confounders C among individuals exposed at the level $A = a^{''}$, to the mediator distribution of individuals exposed at the level $A = a^{'}$.

In practice (when considering A, M_1, M_2, Y binary), we fit to the observed data an imputation model $g[E(Y|A = a, M_1 = m_1, M_2 = m_2, C = C)]$ to impute the outcome conditional on A, M_1, M_2 and C using a generalized linear regression model. The imputation model is used to complete an expansion of the data, in which (i) each observation in the original data set is repeated twice ii) two variables $A^{'}$ and $A^{''}$ are added, and iii) $A^{'}$ is equal to the observed exposure A and $A^{''}$ is equal to the observed exposure A for the first replication and equal to the opposite of the observed exposure for the second replication (Table 3). Only when $A^{'}$ and $A^{''}$ are equal to the observed exposure A the counterfactual outcome $g\{E[Y(a^{''}, M_1(a^{'}), M_2(a^{'}, M_1(a^{'})))|C = c]\}$ can be estimated from observed data, otherwise $g\{E[Y(a^{''}, M_1(a^{'}), M_2(a^{'}, M_1(a^{'})))|C = c]\}$ can be imputed using the fitted values $\widehat{g}\{E[Y(a^{''}, M_1(a^{'}), M_2(a^{'}, M_1(a^{'})))|C = c]\}$ obtained by the imputation model for the outcome with the exposure set to $a^{''}$, the mediators M_1 and M_2 and the baseline covariates C set to their observed values. The imputed outcome is no longer binary, but is substituted by conditional mean imputations. Finally a natural effects model (7) has to be fitted to the imputed data and the conditional effects can be calculated.

The estimation of the marginal effects can be performed by weighting the marginal version of the natural effects model $g\{E[Y(a^{''}, M_1(a^{'}), M_2(a^{'}, M_1(a^{'})))]\} = \theta H(a^{''}, a^{'})$ by the reciprocal of the conditional probability of the exposure A given the covariates C estimated using a logistic regression. Confidence intervals can be calculated by bootstrapping.

Table 3: Imputation approach: example based on two subjects, one exposed (i=1) and the other unexposed (i=2). Bold quantities indicate the unobserved counterfactual values for each subject. A: exposure of the i-subiect, M_1: first mediator of the i-subiect, M_2: second mediator of the i-subiect, Y: outcome of the i-subiect.

i	A	$A^{'}$	$A^{''}$	$M_1(a^{'})$	$M_2(a^{'}, M_1(a^{'}))$	$Y(a^{''}, M_1(a^{'}, M_2(a^{'}, M_1(a^{'}))$
1	1	1	1	$M_1(1)$	$M_2(1, M_1(1))$	$Y(1, M_1(1), M_2(1, M_1(1)))$
1	1	1	**0**	$M_1(1)$	$M_2(1, M_1(1))$	$\mathbf{Y(0, M_1(1), M_2(1, M_1(1)))}$
2	0	0	0	$M_1(0)$	$M_2(0, M_1(0))$	$Y(0, M_1(0), M_2(0, M_1(0)))$
2	0	0	**1**	$M_1(0)$	$M_2(0, M_1(0))$	$\mathbf{Y(1, M_1(0), M_2(0, M_1(0)))}$

Extended imputation approach

The *extended imputation approach* estimates both the marginal and conditional direct and indirect effects by further decomposing the indirect effect into the effect mediated through M_1 and the effect mediated through M_2 alone. Considering conditional effects, the nested counterfactual $g\{E[Y(a^{'''}, M_1(a^{'}), M_2(a^{''}, M_1(a^{'})))|C = c]\}$ is now defined in terms of three newly defined "exposure" variables $A^{'}$, $A^{''}$ and $A^{'''}$ (defined below) as follows:

$$g\{E[Y(a^{'''}, M_1(a^{'}, M_2(a^{''}, M_1(a^{'})))|C = c]\} = \theta H(a^{'''}, a^{'}, a^{''}, c) \quad (12)$$

where $H(a^{'''}, a^{'}, a^{''}, c)$ is a known vector depending on $a^{'''}, a^{'}, a^{''}, c$, and θ is a vector of unknown regression parameters. For example, $H(a^{'''}, a^{'}, a^{''}, c)$ could be $\theta_0 + \theta_1 a^{'''} + \theta_2 a^{'} + \theta_3 a^{''} + \theta_4 a^{'''} a^{'} + \theta_5 a^{'''} a^{''} + \theta_6 a^{'} a^{''} + \theta_7 a^{'''} a^{'} a^{''} + \theta_8 c$. $A^{'}$, $A^{''}$ and $A^{'''}$ are three variables with the same potential levels of A (if A is binary with two levels 0 and 1, then $A^{'}$, $A^{''}$ and $A^{'''}$ have also two hypothetical levels 0 and 1), and their inclusion in the regression model allows to encode the three causal pathways of interest, through neither of the mediators (i.e. the direct pathway $A \to Y$), through M_1 or M_1 and then M_2 (i.e. the indirect pathway through M_1: $A \to M_1 \to Y, A \to M_1 \to M_2 \to Y$) or through M_2 alone (i.e. the partial indirect pathway through M_2: $A \to M_2 \to Y$).

Suppose A is binary, the conditional total effect is equal to:

$$g\{E[Y(1, M_1(1), M_2(1, M_1(1)))|C = c]\} - g\{E[Y(0, M_1(0), M_2(0, M_1(0)))|C = c]\} = \theta_1 + \theta_2 + \theta_3 + \theta_4 + \theta_5 + \theta_6 + \theta_7 \quad (13)$$

the conditional pure direct effect is equal to:

$$g\{E[Y(1, M_1(0), M_2(0, M_1(0)))|C = c]\} - g\{E[Y(0, M_1(0), M_2(0, M_1(0)))|C = c]\} = \theta_1 \quad (14)$$

the conditional total indirect effect through the mediators jointly is equal to:

$$g\{E[Y(1, M_1(1), M_2(1, M_1(1)))|C = c]\} - g\{E[Y(1, M_1(0), M_2(0, M_1(0)))|C = c]\} = \theta_2 + \theta_3 + \theta_4 + \theta_5 + \theta_6 + \theta_7 \quad (15)$$

the conditional total indirect effect through M_1 is equal to:

$$g\{E[Y(1, M_1(1), M_2(1, M_1(1)))|C = c]\} - g\{E[Y(1, M_1(0), M_2(1, M_1(0)))|C = c]\} = \theta_2 + \theta_4 + \theta_6 + \theta_7 \quad (16)$$

and the partial total indirect effect through M_2 is equal to:

$$g\{E[Y(1, M_1(1), M_2(1, M_1(1)))|C = c]\} - g\{E[Y(1, M_1(1), M_2(0, M_1(1)))|C = c]\} = \theta_3 + \theta_5 \quad (17)$$

The nested counterfactual $g\{E[Y(a''', M_1(a'), M_2(a'', M_1(a')))|C = c]\}$ can be estimated from the observed data when a''', a'' and a' equal the observed exposure A. When a''', a'' and a' differ one from others, the nested counterfactual can be estimated according to the following equality:

$$\begin{aligned} g\{E[Y(a''', M_1(a', M_2(a'', M_1(a')))|C = c]\} = \\ = \sum_{m_1, m_2} g[E(Y|A = a''', M_1 = m_1, M_2 = m_2, C = c)] P(M_1 = m_1|A = a', C = c) \\ P(M_2 = m_2|A = a'', M_1 = m_1, C = c) \end{aligned} \quad (18)$$

which is equal to

$$\sum_{m_1, m_2} g[E(Y|A = a''', M_1 = m_1, M_2 = m_2, C = c)] \frac{P(M_1 = m_1|A = a', C = c)}{P(M_1 = m_1|A = a'', C = c)} P(M_1 = m_1, M_2 = m_2|A = a'', C = c) \quad (19)$$

or

$$\sum_{m_1, m_2} g[E(Y|A = a''', M_1 = m_1, M_2 = m_2, C = c)] \frac{P(M_2 = m_2|M_1 = m_1, A = a'', C = c)}{P(M_2 = m_2|M_1 = m_1, A = a', C = c)} P(M_1 = m_1, M_2 = m_2|A = a', C = c) \quad (20)$$

In practice, we fit to the observed data a model for the probability of either M_1 conditional on A and C or M_2 conditional on M_1, A and C (according to the researchers' preference), and we fit to the observed data an imputation model for the outcome $g[E(Y|A = a, M_1 = m_1, M_2 = m_2, C = C)]$ conditional on A, M_1, M_2 and C using a generalized linear regression model. The imputation model is used to complete the expansion of the data, in which each observation in the original data set is repeated four times and three variables A''', A'' and A'

are added to the original exposure variable A. If we are interested in estimating the expression (19), $A^{'}$ is equal to the observed exposure level for the first two replications and equal to the opposite of the observed exposure for the third and fourth replications, $A^{''}$ is equal to the observed exposure level for all four replications, and $A^{'''}$ is equal to the observed exposure level for the first and third replications and equal to the opposite of the observed exposure for the second and fourth replication (Table 5). Only when $A^{'''}$, $A^{''}$ and $A^{'}$ are equal to the observed exposure A the counterfactual outcome $g\{E[Y(a^{'''},M_1(a^{'}),M_2(a^{''},M_1(a^{'})))|C=c]\}$ can be estimated from the observed data, otherwise $g\{E[Y(a^{'''},M_1(a^{'}),M_2(a^{''},M_1(a^{'})))|C=c]\}$ can be imputed using the fitted values $\widehat{g}\{E[Y(a^{'''},M_1(a^{'}),M_2(a^{''},M_1(a^{'})))|C=c]\}$ obtained on the extended dataset by the imputation model for the outcome with the exposure set to $a^{'''}$, the mediators M_1 and M_2 and the baseline covariates C set to their observed values. Similarly the weights $\frac{P(M_1|A=a^{'},C=c)}{P(M_1|A=a^{''},C=c)}$ can be computed on the extended dataset by the model for the probability of M_1 with the exposure set to $a^{'}$ and the baseline covariates C set to their observed values in the numerator and the exposure and the baseline covariates C set to their observed values at the denominator. If we are interested in estimating the expression (20), $A^{'}$ is equal to the observed exposure level for all four replications, $A^{''}$ is equal to the observed exposure level for the first two replications and the counterfactual exposure $1-A$ for the third and fourth replications, and $A^{'''}$ is equal to the value of the observed exposure A for the first and third replications and of the counterfactual exposure $1-A$ for the second and fourth replication (Table 6). Now the counterfactual outcome $g\{E[Y(a^{'''},M_1(a^{'}),M_2(a^{''},M_1(a^{'})))|C=c]\}$ can be imputed using fitted values $\widehat{g}\{E[Y(a^{'''},M_1(a^{'}),M_2(a^{''},M_1(a^{'})))|C=c]\}$ obtained on the extended dataset by the imputation model for the outcome with the exposure set to $a^{'''}$, the mediators M_1 and M_2 and the baseline covariates C set to their observed values. The weights $\frac{P(M_2|M_1,A=a^{''},C=c)}{P(M_2|M_1,A=a^{'},C=c)}$ can be computed on the extended dataset by the model for the probability of M_2 with the exposure set to $a^{''}$, the mediator M_1 and the baseline covariates C set to their observed values in the numerator and the exposure, the mediator M_1 and the baseline covariates C set to their observed values at the denominator. Finally the natural effects model (12) can be fitted by regressing the imputed outcomes on $a^{'}, a^{''}$ and $a^{'''}$ and the covariates C and weighting by weights described above.
The estimation of the marginal effects can be performed by weighting the marginal version of the natural effects model $g\{E[Y(a^{'''},M_1(a^{'}),M_2(a^{''},M_1(a^{'})))]\} = \theta H(a^{'''},a^{'},a^{''})$ by the reciprocal of the conditional probability of the exposure A given the covariates C estimated using a logistic regression. Confidence intervals can be calculated by bootstrapping.

Table 4: Extended imputation approach (expression (31)): example based on two subjects, one exposed (1) and the other unexposed (2). Bold quantities indicate the unobserved counterfactual values for each subject. A: exposure of the i-subiect, M_1: first mediator of the i-subiect, M_2: second mediator of the i-subiect, Y: outcome of the i-subiect.

i	A	$A^{'}$	$A^{''}$	$A^{'''}$	$M_1(a^{'})$	$M_2(a^{''},M_1(a^{'}))$	$Y(a^{''},M_1(a^{'},M_2(a^{''},M_1(a^{'})))$
1	1	1	1	1	$M_1(1)$	$M_2(1,M_1(1))$	$Y(1,M_1(1),M_2(1,M_1(1)))$
1	1	1	1	**0**	$M_1(1)$	$M_2(1,M_1(1))$	$\mathbf{Y(0,M_1(1),M_2(1,M_1(1)))}$
1	1	**0**	1	1	$\mathbf{M_1(0)}$	$\mathbf{M_2(1,M_1(0))}$	$\mathbf{Y(1,M_1(0),M_2(1,M_1(0)))}$
1	1	**0**	1	**0**	$\mathbf{M_1(0)}$	$\mathbf{M_2(1,M_1(0))}$	$\mathbf{Y(0,M_1(0),M_2(1,M_1(0)))}$
2	0	0	0	0	$M_1(0)$	$M_2(0,M_1(0))$	$Y(0,M_1(0),M_2(0,M_1(0)))$
2	0	0	0	**1**	$M_1(0)$	$M_2(0,M_1(0))$	$\mathbf{Y(1,M_1(0),M_2(0,M_1(0)))}$
2	0	**1**	0	0	$\mathbf{M_1(1)}$	$\mathbf{M_2(0,M_1(1))}$	$\mathbf{Y(0,M_1(1),M_2(0,M_1(1)))}$
2	0	**1**	0	**1**	$\mathbf{M_1(1)}$	$\mathbf{M_2(0,M_1(1))}$	$\mathbf{Y(1,M_1(1),M_2(0,M_1(1)))}$

Table 5: Extended imputation approach (expression (32)): example based on two subjects, one exposed (1) and the other unexposed (2). Bold quantities indicate the unobserved counterfactual values for each subject. A: exposure of the i-subiect, M_1: first mediator of the i-subiect, M_2: second mediator of the i-subiect, Y: outcome of the i-subiect.

i	A	$A^{'}$	$A^{''}$	$A^{'''}$	$M_1(a^{'})$	$M_2(a^{''}, M_1(a^{'}))$	$Y(a^{'''}, M_1(a^{'}, M_2(a^{''}, M_1(a^{'})))$
1	1	1	1	1	$M_1(1)$	$M_2(1, M_1(1))$	$Y(1, M_1(1), M_2(1, M_1(1))$
1	1	1	1	**0**	$M_1(1)$	$M_2(1, M_1(1))$	$\mathbf{Y(0, M_1(1), M_2(1, M_1(1))}$
1	1	1	**0**	1	$M_1(1)$	$\mathbf{M_2(0, M_1(1))}$	$\mathbf{Y(1, M_1(1), M_2(0, M_1(1))}$
1	1	1	**0**	**0**	$M_1(1)$	$\mathbf{M_2(0, M_1(1))}$	$\mathbf{Y(0, M_1(1), M_2(0, M_1(1))}$
2	0	0	0	0	$M_1(0)$	$M_2(0, M_1(0))$	$Y(0, M_1(0), M_2(0, M_1(0))$
2	0	0	0	**1**	$M_1(0)$	$M_2(0, M_1(0))$	$\mathbf{Y_2(1, M_1(0), M_2(0, M_1(0))}$
2	0	0	**1**	0	$M_1(0)$	$\mathbf{M_2(1, M_1(0))}$	$\mathbf{Y(0, M_1(0), M_2(1, M_1(0))}$
2	0	0	**1**	**1**	$M_1(0)$	$\mathbf{M_2(1, M_1(0))}$	$\mathbf{Y(1, M_1(0), M_2(1, M_1(0))}$

Summary of the fitted regression models to implement each of the four approaches to sequential mediation analysis

Inverse odds ratio weighting approach. To estimate the conditional total effect we fitted a Poisson regression model to the probability of the occurrence of wheezing between 6 and 18 months of infant life (Y) conditional on depression or anxiety in pregnancy (A) and maternal age, education, residence and body mass index at the beginning of pregnancy, parity and child's sex (C). We assumed no interactions between A and C. To estimate the weights (9), we fitted a logistic regression model of depression or anxiety in pregnancy (A) conditional on adverse reproductive outcomes (M_1), occurrence of lower respiratory infection (M_2) and maternal age, education, residence and body mass index at the beginning of pregnancy, parity and child's sex (C). We included a term for the interaction betweenM_1 and M_2. Percentiles confidence intervals were calculated by bootstrap with 1000 repetitions.

Inverse probability weighting approach. To estimate the probability of occurrence of wheezing between 6 and 18 months of infant life (Y), we fitted a Poisson regression model conditional on depression or anxiety in pregnancy (A), adverse reproductive outcomes (M_1), occurrence of lower respiratory infections (M_2) and maternal age, education, residence and body mass index at the beginning of pregnancy, parity and child's sex (C). We included terms for the interactions between M_1 and M_2. To estimate the weights in the same expressions, we fitted a logistic regression model on depression or anxiety in pregnancy (A) conditional on maternal age, education, residence and body mass index at the beginning of the pregnancy, parity and child's sex (C). We assumed no interactions between A and C. Percentiles confidence intervals were calculated by bootstrap with 1000 repetitions.

Imputation-based approach. To estimate the imputed nested counterfactuals, we fitted a Poisson regression model to the probability of occurrence of wheezing between 6 and 18 months of infant life (Y) conditional on depression or anxiety in pregnancy (A), adverse reproductive outcomes (M_1), occurrence of lower respiratory infections (M_2) and maternal age, education, residence and body mass index at the beginning of pregnancy, parity and child's sex (C). We included terms for the interactions between M_1 and M_2. To estimate the conditional total, direct and indirect effects we fitted a Poisson regression model to the imputed nested counterfactuals conditional on the newly created variables for maternal depression or anxiety in pregnancy ($A^{'}$ and $A^{''}$) and maternal age, education, residence and body mass index at the beginning of the pregnancy, parity and child's sex (C). Similarly, to estimate the marginal total, direct and indirect effects we first calculated the weights $P(A|C)$ using a logistic regression model on depression or anxiety in pregnancy (A) conditional on maternal age, education, residence and body mass index at the beginning of the pregnancy, parity and child's sex (C), and then fitted a Poisson regression

model conditional on the newly created variables for maternal depression or anxiety in pregnancy ($A^{'}$ and $A^{''}$) and weighted by the inverse of the estimated probability of A conditional on C. We assumed no interactions between A and C. Percentiles confidence intervals were calculated by bootstrap with 1000 repetitions.

Extended imputation-based approach. To estimate the imputed nested counterfactuals, we fitted a Poisson regression model to the probability of occurrence of wheezing between 6 and 18 months of infant life (Y) conditional on depression or anxiety in pregnancy (A), adverse reproductive outcomes (M_1), occurrence of lower respiratory infections (M_2) and maternal age, education, residence and body mass index at the beginning of the pregnancy, parity and child's sex (C). We included terms for the interactions between M_1 and M_2. To estimate the weights on M_1, we fitted a Poisson regression model to the probability of the adverse reproductive outcomes (M_1) conditional on the newly created variables for maternal depression or anxiety in pregnancy ($A^{'}$ and $A^{''}$) and maternal age, education, residence and body mass index at the beginning of the pregnancy, parity and child's sex (C). To estimate the weights on M_2, we fitted a Poisson regression model to the probability of the occurrence of lower respiratory infection (M_2) conditional on adverse reproductive outcomes (M_1) and the newly created variables for maternal depression or anxiety in pregnancy ($A^{'}$ and $A^{''}$) and maternal age, education, residence and body mass index at the beginning of the pregnancy, and parity (C). To estimate the conditional total, joint direct and joint and partial indirect effects, we fitted a Poisson regression model to the imputed nested counterfactuals conditional on the newly created variables for maternal depression or anxiety in pregnancy ($A^{'}, A\prime\prime$ and $A\prime\prime\prime$) and maternal age, education, residence and body mass index at the beginning of the pregnancy, parity and child's sex (C), weighted by the weights described above. The marginal effects were calculated by further weighting by the inverse of the probability of A conditional on C estimated using a logistic regression. Percentiles confidence intervals were calculated by bootstrap with 1000 repetitions.

R Code for multiple mediation analysis

```
##########################################################
  # mydata is a dataframe with the following columns:
  # a is the binary exposure
  #m1 is the first binary mediator
  #m2 is the second binary mediator
  #c is a vector of potential baseline confounders (11 elements in our case-study)
  #y is the binary outcome

##########################################################
library(MASS)
library(boot)
##########################################################
###Inverse odds ratio weighting
##########################################################
###################
# estimation procedure
###################
  theta= function(mydata,indices) {
  d=mydata[indices,]
  # model for the exposure
  fit.a=glm(formula = a ~ m1*m2+c, family = "binomial", data = d)
  predprob=predict(fit.a, d, type="response")
```

```
  inverseodds=((1-predprob)/predprob)
  # calculate weights
  wt_iow=numeric()
  wt_iow[d$a==0]=1
  wt_iow[d$a==1]=inverseodds[d$a==1]
  # total effect
  fit.y=glm(formula = y ~ a+c, family = "poisson", data = d)
  tot=exp(coef(fit.y))[2]
  # direct effect
  fit.y.weighted=glm(formula = y ~ a+c, weights=wt_iow, family = "poisson", data = d)
  pde=exp(coef(fit.y.weighted))[2]
  # indirect effect
  tie=tot/pde
  return(c(pde,tie,tot))
}
###################
#bootstrap of the estimation procedure
###################
replicates=1000
boot.se <- boot(data=mydata, statistic=theta, R=replicates)
pde=matrix(,nrow=1,ncol=3)
tie=matrix(,nrow=1,ncol=3)
tot=matrix(,nrow=1,ncol=3)
interval_conf=boot.ci(boot.se, type="perc", index=1)
pde[1]=boot.se$t0[1]
pde[2]=interval_conf$percent[4]
pde[3]=interval_conf$percent[5]
interval_conf=boot.ci(boot.se, type="perc", index=2)
tie[1]=boot.se$t0[2]
tie[2]=interval_conf$percent[4]
tie[3]=interval_conf$percent[5]
interval_conf=boot.ci(boot.se, type="perc", index=3)
tot[1]=boot.se$t0[3]
tot[2]=interval_conf$percent[4]
tot[3]=interval_conf$percent[5]
pde
tie
tot

##########################################################
###Inverse probability weighting
##########################################################
##########################################################
# estimation procedure without interaction between A and M1 and M2
##########################################################
theta= function(mydata,indices) {
  d=mydata[indices,]
```

```
  # compute the weights
  logit=glm(formula = a ~ c, family = "binomial", data = d)
  prob=data.frame(predict(logit, d, type="response"))
  colnames(prob)[1]="p"
  prob$pdm=prob$p
  prob$pdm[d$a==0]=1-prob$p[d$a==0]
  prob$w=1/prob$pdm
  # model the outcome
  fit=glm(formula = y ~ a+m1*m2+c, family = "poisson", data = d)
  #predict the outcome forcing A=0
   pred.frame0=d
   pred.frame0$a=0
   p0=data.frame(predict(fit,se.fit=TRUE,newdata=pred.frame0,type="response"))
   # predict the outcome forcing A=1
   pred.frame1=d
   pred.frame1$a=1
   p1=predict(fit,se.fit=TRUE,newdata=pred.frame1,type="response")
   # E[Y0M0]
   p00=weighted.mean(p0$fit[d$a==0],prob$w[d$a==0], na.rm = TRUE)
   # E[Y1M1]
   p11=weighted.mean(p1$fit[d$a==1],prob$w[d$a==1], na.rm = TRUE)
   # E[Y1M0]
   p10=weighted.mean(p1$fit[d$a==0],prob$w[d$a==0], na.rm = TRUE)
   # compute the effects estimate
   pde=p10/p00
   tie=p11/p10
   tot=pde*tie
   return(c(pde,tie,tot))
}
###########################################################
# estimation procedure with interaction between A and M1 and M2
###########################################################
theta= function(mydata,indices) {
  d=mydata[indices,]
  # compute the weights
  logit=glm(formula = a ~ c, family = "binomial", data = d)
  prob=data.frame(predict(logit, d, type="response"))
  colnames(prob)[1]="p"
  prob$pdm=prob$p
  prob$pdm[d$a==0]=1-prob$p[d$a==0]
  prob$w=1/prob$pdm
  # model the outcome
  fit=glm(formula = y ~ a*m1*m2+m1*m2+c, family = "poisson", data = d)
  #predict the outcome forcing A=0
   pred.frame0=d
   pred.frame0$a=0
   p0=data.frame(predict(fit,se.fit=TRUE,newdata=pred.frame0,type="response"))
```

```
   # predict the outcome forcing A=1
   pred.frame1=d
   pred.frame1$a=1
   p1=predict(fit,se.fit=TRUE,newdata=pred.frame1,type="response")
   # E[Y0M0]
   p00=weighted.mean(p0$fit[d$a==0],prob$w[d$a==0], na.rm = TRUE)
   # E[Y1M1]
   p11=weighted.mean(p1$fit[d$a==1],prob$w[d$a==1], na.rm = TRUE)
   # E[Y1M0]
   p10=weighted.mean(p1$fit[d$a==0],prob$w[d$a==0], na.rm = TRUE)
   # compute the effects estimate
   pde=p10/p00
   tie=p11/p10
   tot=pde*tie
   return(c(pde,tie,tot))
}
###################
#bootstrap of the estimation procedure
###################
replicates=1000
boot.se <- boot(data=mydata, statistic=theta, R=replicates)
pde=matrix(,nrow=1,ncol=3)
tie=matrix(,nrow=1,ncol=3)
tot=matrix(,nrow=1,ncol=3)
interval_conf=boot.ci(boot.se, type="perc", index=1)
pde[1]=boot.se$t0[1]
pde[2]=interval_conf$percent[4]
pde[3]=interval_conf$percent[5]
pde
interval_conf=boot.ci(boot.se, type="perc", index=2)
tie[1]=boot.se$t0[2]
tie[2]=interval_conf$percent[4]
tie[3]=interval_conf$percent[5]
tie
interval_conf=boot.ci(boot.se, type="perc", index=3)
tot[1]=boot.se$t0[3]
tot[2]=interval_conf$percent[4]
tot[3]=interval_conf$percent[5]
tot
##########################################################
###Imputation-based approach
##########################################################
library(medflex)
##########################################################
# estimation procedure and bootstrap without interaction between A and
#M1 and M2 for conditional effects
##########################################################
```

```
impdata<-neImpute(y ~ as.factor(a)+m1*m2+c, family = poisson,  data = mydata, nMed=2)
head(impdata)
nemod=neModel(y ~ a0+a1+c, family = "poisson", expData = impdata,se="bootstrap",nBoot = 1000)
summary(nemod)
lht<-neLht(nemod,linfct=c("a01=0","a11=0", "a01+a11=0"))
exp(cbind(coef(lht),confint(lht,type="perc"))) #confint with bootstrap
##########################################################
# estimation procedure and bootstrap without interaction between A and
#M1 and M2 for marginal effects
##########################################################
expfit<-glm(a ~ c, data = mydata, family="binomial")
impdata<-neImpute(y ~ as.factor(a)+m1*m2+c, family = "poisson", data = mydata, nMed=2)
nemod=neModel(y ~ a0+a1, family = "poisson", xFit=expfit, expData = impdata, se="bootstrap",
nBoot = 1000)
summary(nemod)
lht<-neLht(nemod,linfct=c("a01=0","a11=0", "a01+a11=0"))
exp(cbind(coef(lht),confint(lht,type="perc")))

##########################################################
# estimation procedure and bootstrap with interaction between A and M1
#and M2 for conditional effects
##########################################################
impdata<-neImpute(y ~ as.factor(a)*m1*m2+m1*m2+c, family = poisson,  data = mydata, nMed=2)
nemod=neModel(y ~ a0*a1+c, family = "poisson", expData = impdata,se="bootstrap",nBoot = 1000)
summary(nemod)
lht<-neLht(nemod,linfct=c("a01=0","a11+a01:a11=0", "a01+a11+a01:a11=0"))
exp(cbind(coef(lht),confint(lht,type="perc")))
##########################################################
# estimation procedure and bootstrap with interaction between A and M1
#and M2 for marginal effects
##########################################################
expfit<-glm(a ~ c, data = mydata, family="binomial")
impdata<-neImpute(y ~ as.factor(a)*m1*m2+m1*m2+c, family = "poisson", data = mydata,nMed=2)
nemod=neModel(y ~ a0*a1, family = "poisson", xFit=expfit, expData = impdata, se="bootstrap",
nBoot = 1000)
summary(nemod)
lht<-neLht(nemod,linfct=c("a01=0","a11+a01:a11=0", "a01+a11+a01:a11=0"))
exp(cbind(coef(lht),confint(lht,type="perc")))
##########################################################
#######Extended imputation-based approach
##########################################################
library(reshape)
mydata<-mydata[order(mydata$id_characteristic),]
##########################################################
# estimation procedure and bootstrap without interaction between A and
#M1 and M2 for conditional effects
##########################################################
```

```
theta <- function(data, index) {
  dat <- data[index, ]
  #Fit a model for the probability of the two mediators conditional on A,
  #potential earlier intermediates and C
  fitM1<-glm(m1 ~ as.factor(a)+c,   family = "poisson", data = dat)
  fitM2<-glm(m2 ~ as.factor(a)+m1+c, family = "poisson", data = mydata)
 #Fit a model for the outcome mean conditional on A, M1, M2 and C
  fitY<-glm(y ~ as.factor(a)+m1*m2+c, family = "poisson", data = dat)
#Extend the data set and create auxiliary variables a1, a2, a3
 ex<-untable(dat,num=4)
 ex<-ex[order(ex$id_characteristic),]
 extdat <- data.frame(replicate = rep(1:4, times = nrow(dat)), ex,a3 = NA, a1 = NA, a2 = NA)
 levels<-c(0,1)
 tmp<-lapply(dat$id_characteristic,function(x) expand.grid(rep(list(levels),2)))
  tmp<-do.call(rbind,tmp)
  extdat1<-within(extdat,{
         a3<-as.factor(tmp$Var1)
    a1<-as.factor(tmp$Var2)
   a2<-as.factor(extdat$a)
  })
   extdat2<-within(extdat,{
        a1<-as.factor(extdat$a)
        a2<-as.factor(tmp$Var2)
        a3<-as.factor(tmp$Var1)
    })
 #Calculate weights: W1=P(M1 | A=a1,C)/P(M1 | A=a2,C)
  num1 <- with(extdat1,predict(fitM1,newdata=within(extdat1,{a=a1}),type="response"))
  denom1 <- with(extdat1,predict(fitM1,newdata=within(extdat1,{a=a2}),type="response"))
  extdat1$W1 <- num1/denom1
  #Calculate weights: W2=P(M2 | A=a2,C)/P(M2 | A=a1,C)
  num2<-with(extdat2,predict(fitM2,newdata=within(extdat2,{a=a2}),type="response"))
  denom2<-with(extdat2,predict(fitM2,newdata=within(extdat2,{a=a1}),type="response"))
  extdat2$W2<-num2/denom2
  #impute nested counterfactual Y(a,M1(a1),M2(a2,M1(a1)))
  extdat1$Y<-predict(fitY,newdata=within(extdat1,{a<-a3}),type="response")
  extdat2$Y<-predict(fitY,newdata=within(extdat2,{a<-a3}),type="response")
  #fit a natural effect model for g(E(Y(a,M1(a1),M2(a2,M1(a1)))))
  fitNEM1<-glm(Y ~ a3+a1+a2+c, family = "poisson", data = extdat1, weights=W1)
  fitNEM2<-glm(Y ~ a3+a1+a2+c, family = "poisson", data = extdat2, weights=W2)
  return(coef(fitNEM1))
  return(coef(fitNEM2))
}

###################
#bootstrap of the estimation procedure
###################
replicates=1000
```

```
bootSE <- boot(data=mydata, statistic=theta, R=replicates)
# function to calculate 95% percentiles confidence intervals
var<-numeric()
linfunCI.perc <- function(boot.out, L, conf) {
est <- sum(L %*% boot.out$t0)
for (i in 1:replicates){
var[i] <- sum(L %*% boot.out$t[i,])
}
  upCI <- quantile(var,c(0.975))
  lowCI <- quantile(var,c(0.025))
  return(exp(c(est,lowCI,upCI)))
}
#joint natural direct effect:theta1
L <- c(0, 1, 0, 0, 0, 0, 0, 0, 0, 0, 0, 0, 0, 0, 0)
pde<-linfunCI.perc(bootSE, L, 0.95)
#joint natural indirect effect:theta2+theta3
L <- c(0, 0, 1, 1, 0, 0, 0, 0, 0, 0, 0, 0, 0, 0, 0)
nie<-linfunCI.perc(bootSE, L, 0.95)
#natural indirect effect by M1:theta2
L <- c(0, 0, 1, 0, 0, 0, 0, 0, 0, 0, 0, 0, 0, 0, 0)
iem1<-linfunCI.perc(bootSE, L, 0.95)
#partial indirect effect by M2:theta3
L <- c(0, 0, 0, 1, 0, 0, 0, 0, 0, 0, 0, 0, 0, 0, 0)
iem2<-linfunCI.perc(bootSE, L, 0.95)
  #total effect
L <- c(0, 1,  1, 1, 0, 0, 0, 0, 0, 0, 0, 0, 0, 0, 0)
te<-linfunCI.perc(bootSE, L, 0.95)
pde
nie
iem1
iem2
te
##########################################################
# estimation procedure and bootstrap without interaction between A and
#M1 and M2 for marginal effects
##########################################################
theta <- function(data, index) {
  dat <- data[index, ]
  #Fit a model for the probability of A conditional on C
  fitA<-glm(a ~ c,   family = "binomial", data = dat)
  #Fit a model for the probability of the two mediators conditional on A,
  #potential earlier intermediates and C
  fitM1<-glm(m1 ~ as.factor(a)+c,   family = "poisson", data = dat)
  fitM2<-glm(m2 ~ as.factor(a)+m1+c, family = "poisson", data = mydata)
 #Fit a model for the outcome mean conditional on A, M1, M2 and C
  fitY<-glm(y ~ as.factor(a)+m1*m2+c, family = "poisson", data = dat)
#Extend the data set and create auxiliary variables a1, a2, a3
```

```
 ex<-untable(dat,num=4)
 ex<-ex[order(ex$id_characteristic),]
 extdat <- data.frame(replicate = rep(1:4, times = nrow(dat)), ex,a3 = NA, a1 = NA, a2 = NA)
 levels<-c(0,1)
 tmp<-lapply(dat$id_characteristic,function(x) expand.grid(rep(list(levels),2)))
  tmp<-do.call(rbind,tmp)
  extdat1<-within(extdat,{
         a3<-as.factor(tmp$Var1)
    a1<-as.factor(tmp$Var2)
   a2<-as.factor(extdat$a)
  })
   extdat2<-within(extdat,{
        a1<-as.factor(extdat$a)
        a2<-as.factor(tmp$Var2)
        a3<-as.factor(tmp$Var1)
    })
 #Calculate weights: W1=P(M1 | A=a1,C)/P(M1 | A=a2,C)
  num1 <- with(extdat1,predict(fitM1,newdata=within(extdat1,{a=a1}),type="response"))
  denom1 <- with(extdat1,predict(fitM1,newdata=within(extdat1,{a=a2}),type="response"))
  extdat1$W1 <- num1/denom1
  #Calculate weights: W2=P(M2 | A=a2,C)/P(M2 | A=a1,C)
  num2<-with(extdat2,predict(fitM2,newdata=within(extdat2,{a=a2}),type="response"))
  denom2<-with(extdat2,predict(fitM2,newdata=within(extdat2,{a=a1}),type="response"))
  extdat2$W2<-num2/denom2
  #impute nested counterfactual Y(a,M1(a1),M2(a2,M1(a1)))
  extdat1$Y<-predict(fitY,newdata=within(extdat1,{a<-a3}),type="response")
  extdat2$Y<-predict(fitY,newdata=within(extdat2,{a<-a3}),type="response")
 #re-calculate the weights by weighting for the inverse of the probability of A
 extdat1<-within(extdat1,{W1<-W1/dbinom(a,size=1,prob=predict(fitA,newdata=extdat1,
 type="response"))})
  extdat2<-within(extdat2,{W2<-W2/dbinom(a,size=1,prob=predict(fitA,newdata=extdat2,
  type="response"))})
 #fit a natural effect model for g(E(Y(a,M1(a1),M2(a2,M1(a1))))
  fitNEM1<-glm(Y ~ a3+a1+a2, family = "poisson", data = extdat1, weights=W1)
  fitNEM2<-glm(Y ~ a3+a1+a2, family = "poisson", data = extdat2, weights=W2)
  return(coef(fitNEM1))
  return(coef(fitNEM2))
}
###################
#bootstrap of the estimation procedure
###################
replicates=1000
bootSE <- boot(data=mydata, statistic=theta, R=replicates)
# function to calculate 95% percentiles confidence intervals
var<-numeric()
linfunCI.perc <- function(boot.out, L, conf) {
est <- sum(L %*% boot.out$t0)
```

```
for (i in 1:replicates){
var[i] <- sum(L %*% boot.out$t[i,])
}
  upCI <- quantile(var,c(0.975))
  lowCI <- quantile(var,c(0.025))
  return(exp(c(est,lowCI,upCI)))
}
#joint natural direct effect:theta1
L <- c(0, 1, 0, 0)
pde<-linfunCI.perc(bootSE, L, 0.95)
#joint natural indirect effect:theta2+theta3
L <- c(0, 0, 1, 1)
nie<-linfunCI.perc(bootSE, L, 0.95)
#natural indirect effect by M1:theta2
L <- c(0, 0, 1, 0)
iem1<-linfunCI.perc(bootSE, L, 0.95)
#partial indirect effect by M2:theta3
L <- c(0, 0, 0, 1)
iem2<-linfunCI.perc(bootSE, L, 0.95)
  #total effect
L <- c(0, 1,  1, 1)
te<-linfunCI.perc(bootSE, L, 0.95)
pde
nie
iem1
iem2
te
##########################################################
# estimation procedure and bootstrap with interaction between A and M1
#and M2 for conditional effects
##########################################################
theta <- function(data, index) {
  dat <- data[index, ]
  #Fit a model for the probability of the two mediators conditional on A,
  #potential earlier intermediates and C
  fitM1<-glm(m1 ~as.factor(a)+c,   family = "poisson", data = dat)
  fitM2<-glm(m2 ~ as.factor(a)*m1+c, family = "poisson", data = mydata)
 #Fit a model for the outcome mean conditional on A, M1, M2 and C
  fitY<-glm(y ~ as.factor(a)*m1*m2+m1*m2+c, family = "poisson", data = dat)
#Extend the data set and create auxiliary variables a1, a2, a3
 ex<-untable(dat,num=4)
 ex<-ex[order(ex$id_characteristic),]
 extdat <- data.frame(replicate = rep(1:4, times = nrow(dat)), ex,a3 = NA, a1 = NA, a2 = NA)
 levels<-c(0,1)
 tmp<-lapply(dat$id_characteristic,function(x) expand.grid(rep(list(levels),2)))
  tmp<-do.call(rbind,tmp)
  extdat1<-within(extdat,{
```

```
         a3<-as.factor(tmp$Var1)
    a1<-as.factor(tmp$Var2)
   a2<-as.factor(extdat$a)
  })
   extdat2<-within(extdat,{
        a1<-as.factor(extdat$a)
        a2<-as.factor(tmp$Var2)
        a3<-as.factor(tmp$Var1)
    })
 #Calculate weights: W1=P(M1 | A=a1,C)/P(M1 | A=a2,C)
  num1 <- with(extdat1,predict(fitM1,newdata=within(extdat1,{a=a1}),type="response"))
  denom1 <- with(extdat1,predict(fitM1,newdata=within(extdat1,{a=a2}),type="response"))
  extdat1$W1 <- num1/denom1
  #Calculate weights: W2=P(M2 | A=a2,C)/P(M2 | A=a1,C)
  num2<-with(extdat2,predict(fitM2,newdata=within(extdat2,{a=a2}),type="response"))
  denom2<-with(extdat2,predict(fitM2,newdata=within(extdat2,{a=a1}),type="response"))
  extdat2$W2<-num2/denom2
  #impute nested counterfactual Y(a,M1(a1),M2(a2,M1(a1)))
  extdat1$Y<-predict(fitY,newdata=within(extdat1,{a<-a3}),type="response")
  extdat2$Y<-predict(fitY,newdata=within(extdat2,{a<-a3}),type="response")
  #fit a natural effect model for g(E(Y(a,M1(a1),M2(a2,M1(a1))))
  fitNEM1<-glm(Y ~ a3*a1*a2+c, family = "poisson", data = extdat1, weights=W1)
  fitNEM2<-glm(Y ~ a3*a1*a2+c, family = "poisson", data = extdat2, weights=W2)
   return(coef(fitNEM1))
  return(coef(fitNEM2))
}
###################
#bootstrap of the estimation procedure
###################

replicates=1000
bootSE <- boot(data=mydata, statistic=theta, R=replicates)
# function to calculate 95% percentiles confidence intervals
var<-numeric()
linfunCI.perc <- function(boot.out, L, conf) {
est <- sum(L %*% boot.out$t0)
for (i in 1:replicates){
var[i] <- sum(L %*% boot.out$t[i,])
}
  upCI <- quantile(var,c(0.975))
  lowCI <- quantile(var,c(0.025))
  return(exp(c(est,lowCI,upCI)))
}
#joint natural direct effect:theta1
L <- c(0, 1, 0, 0, 0, 0, 0, 0, 0, 0, 0, 0, 0, 0, 0, 0, 0, 0, 0)
pde<-linfunCI.perc(bootSE, L, 0.95)
#joint natural indirect effect:theta2+theta3+theta16+theta17+theta18+theta19
```

```
L <- c(0, 0, 1, 1, 0, 0, 0, 0, 0, 0, 0, 0, 0, 0, 0, 1, 1, 1, 1)
nie<-linfunCI.perc(bootSE, L, 0.95)
#natural indirect effect by M1:theta2+theta16+theta18+theta19
L <- c(0, 0, 1, 0, 0, 0, 0, 0, 0, 0, 0, 0, 0, 0, 0, 1, 0, 1, 1)
iem1<-linfunCI.perc(bootSE, L, 0.95)
#partial indirect effect by M2:theta3+theta17
L <- c(0, 0, 0, 1, 0, 0, 0, 0, 0, 0, 0, 0, 0, 0, 0, 0, 1, 0, 0)
iem2<-linfunCI.perc(bootSE, L, 0.95)
  #total effect
L <- c(0, 1,  1, 1, 0, 0, 0, 0, 0, 0, 0, 0, 0, 0, 0, 1, 1, 1, 1)
te<-linfunCI.perc(bootSE, L, 0.95)
pde
nie
iem1
iem2
te
##########################################################
# estimation procedure and bootstrap with interaction between A and M1
#and M2 for marginal effects
##########################################################
theta <- function(data, index) {
  dat <- data[index, ]
  #Fit a model for the probability of A conditional on C
  fitA<-glm(a ~ c,   family = "binomial", data = dat)
  #Fit a model for the probability of the two mediators conditional on A,
  #potential earlier intermediates and C
  fitM1<-glm(m1 ~ as.factor(a)+c,   family = "poisson", data = dat)
  fitM2<-glm(m2 ~ as.factor(a)*m1+c, family = "poisson", data = mydata)
 #Fit a model for the outcome mean conditional on A, M1, M2 and C
  fitY<-glm(y ~ as.factor(a)*m1*m2+m1*m2+c, family = "poisson", data = dat)
#Extend the data set and create auxiliary variables a1, a2, a3
 ex<-untable(dat,num=4)
 ex<-ex[order(ex$id_characteristic),]
 extdat <- data.frame(replicate = rep(1:4, times = nrow(dat)), ex,a3 = NA, a1 = NA, a2 = NA)
 levels<-c(0,1)
 tmp<-lapply(dat$id_characteristic,function(x) expand.grid(rep(list(levels),2)))
  tmp<-do.call(rbind,tmp)
  extdat1<-within(extdat,{
         a3<-as.factor(tmp$Var1)
    a1<-as.factor(tmp$Var2)
   a2<-as.factor(extdat$a)
  })
   extdat2<-within(extdat,{
        a1<-as.factor(extdat$a)
        a2<-as.factor(tmp$Var2)
        a3<-as.factor(tmp$Var1)
    })
```

```
 #Calculate weights: W1=P(M1 | A=a1,C)/P(M1 | A=a2,C)
  num1 <- with(extdat1,predict(fitM1,newdata=within(extdat1,{a=a1}),type="response"))
  denom1 <- with(extdat1,predict(fitM1,newdata=within(extdat1,{a=a2}),type="response"))
  extdat1$W1 <- num1/denom1
  #Calculate weights: W2=P(M2 | A=a2,C)/P(M2 | A=a1,C)
  num2<-with(extdat2,predict(fitM2,newdata=within(extdat2,{a=a2}),type="response"))
  denom2<-with(extdat2,predict(fitM2,newdata=within(extdat2,{a=a1}),type="response"))
  extdat2$W2<-num2/denom2
  #impute nested counterfactual Y(a,M1(a1),M2(a2,M1(a1)))
  extdat1$Y<-predict(fitY,newdata=within(extdat1,{a<-a3}),type="response")
  extdat2$Y<-predict(fitY,newdata=within(extdat2,{a<-a3}),type="response")
 #re-calculate the weights by weighting for the inverse of the probability of A
  extdat1<-within(extdat1,{W1<-W1/dbinom(a,size=1,prob=predict(fitA,newdata=extdat1,
  type="response"))})
  extdat2<-within(extdat2,{W2<-W2/dbinom(a,size=1,prob=predict(fitA,newdata=extdat2,
  type="response"))})
  #fit a natural effect model for g(E(Y(a,M1(a1),M2(a2,M1(a1))))
  fitNEM1<-glm(Y ~ a3*a1*a2, family = "poisson", data = extdat1, weights=W1)
  fitNEM2<-glm(Y ~ a3*a1*a2, family = "poisson", data = extdat2, weights=W2)
  return(coef(fitNEM1))
  return(coef(fitNEM2))
}
###################
#bootstrap of the estimation procedure
###################
replicates=1000
bootSE <- boot(data=mydata, statistic=theta, R=replicates)
# function to calculate 95% percentiles confidence intervals
var<-numeric()
linfunCI.perc <- function(boot.out, L, conf) {
est <- sum(L %*% boot.out$t0)
for (i in 1:replicates){
var[i] <- sum(L %*% boot.out$t[i,])
}
  upCI <- quantile(var,c(0.975))
  lowCI <- quantile(var,c(0.025))
  return(exp(c(est,lowCI,upCI)))
}
#joint natural direct effect:theta1
L <- c(0, 1, 0, 0, 0, 0, 0, 0)
pde<-linfunCI.perc(bootSE, L, 0.95)
#joint natural indirect effect:theta2+theta3+theta4+theta5+theta6+theta7
L <- c(0, 0, 1, 1, 1, 1, 1, 1)
nie<-linfunCI.perc(bootSE, L, 0.95)
#natural indirect effect by M1:theta2+theta4+theta6+theta7
L <- c(0, 0, 1, 0, 1, 0, 1,1)
iem1<-linfunCI.perc(bootSE, L, 0.95)
```

```
#partial indirect effect by M2:theta3+theta5
L <- c(0, 0, 0, 1, 0, 1, 0, 0)
iem2<-linfunCI.perc(bootSE, L, 0.95)
  #total effect
L <- c(0, 1,  1, 1,  1, 1, 1, 1)
te<-linfunCI.perc(bootSE, L, 0.95)
pde
nie
iem1
iem2
te
```
